# A Framing Analysis of Consultation Submissions on the WHO Global Strategy to Reduce the Harmful Use of Alcohol: Values and Interests

**DOI:** 10.34172/ijhpm.2021.68

**Published:** 2021-06-26

**Authors:** Chiara Rinaldi, May CI van Schalkwyk, Matt Egan, Mark Petticrew

**Affiliations:** ^1^Department of Health Services Research and Policy, London School of Hygiene and Tropical Medicine, London, UK.; ^2^Department of Public Health, Environments and Society, London School of Hygiene and Tropical Medicine, London, UK.

**Keywords:** Framing Analysis, Alcohol Policy, Alcohol Industy, World Health Organization

## Abstract

**Background:** In response to the magnitude of harms caused by alcohol, the World Health Organization (WHO) Global Strategy to Reduce the Harmful Use of Alcohol (GAS) was endorsed in 2010. We analysed submissions to the 2019 WHO consultation on the implementation of the GAS to identify how different stakeholders frame alcohol use and control; and to assess how stakeholders engage with the consultation process, with possibly harmful consequences for public health policy.

**Methods:** All submissions from WHO Member States, international organisations, non-governmental organisations (NGOs), academic institutions and private sector entities were identified and used as data for an inductive framing analysis. This involved close reading and data familiarisation, thematic coding and identifying emergent framings. Through the analysis of texts, framing analysis can give insights into the values and interests of stakeholders. Because framing influences how issues are conceptualised and addressed, framing analysis is a useful tool to study policy-making processes.

**Results:** We identified 161 unique submissions and seven attachments. Emerging frames were grouped according to their function: defining the problem, assigning causation, proposing solutions, or justifying and persuading. Submissions varied in terms of the framing they deployed and how this was presented, eg, how the problem was defined. Proposed policy solutions also varied. Targeted solutions emphasising individual responsibility tended to be supported by industry and some Member States. Calls for universal regulation and global mobilisation often came from NGOs and academia. Stakeholders drew on evidence and specific value systems to support the adoption of certain problem and solution ideas and to oppose competing framing.

**Conclusion:** Alcohol control is a contested policy field in which different stakeholders use framing to set the agenda and influence what policy solutions are considered legitimate. WHO should consider which interests are served by these different framings and how to weigh different stakeholders in the consultation process.

## Background

**Key Messages**

**Implications for policy makers**

- Alcohol harms are conceptualised differently by stakeholders in different sectors, using different framing, which has consequences for how the issue is understood and what action is advocated to prevent and control the harmful use of alcohol.
- Private sector entities and some Member States predominantly framed alcohol harms as a problem of individual responsibility with largely individual consequences, therefore requiring targeted interventions instead of population-level regulation.
- Most Member States, international organisations, non-governmental organisations (NGOs) and academic institutions who engaged with the consultation framed alcohol harms as a collective problem with wide-ranging harmful consequences beyond health, thus requiring regulation and national (eg, ‘best buys’) and global level (eg, binding treaty).
- Some private sector entities appeared to reject the notion of inherent conflicts of interest between public health and the alcohol industry, while most stakeholders in other sectors called for restrictions on industry interference in alcohol policy given the evidence of industry attempts at policy influence or dilution.
- The values and interests underlying the identified framings should be taken into account by World Health Organization (WHO) when assessing policy proposals. The role that is given to alcohol industry stakeholders in dialogue and consultations with the WHO should be carefully considered.

**Implications for the public**

Alcohol use is a significant risk factor for deaths and ill-health worldwide and is therefore high on the public health agenda, exemplified by the adoption of the World Health Organization (WHO) Global Strategy to Reduce the Harmful Use of Alcohol (GAS). This analysis of submissions to a consultation on the WHO GAS reveals that stakeholders in the alcohol policy-making process have different views on the problem of harmful use of alcohol, its causes and potential solutions. Divergence is especially prominent between private sector entities with commercial interests in alcohol production and some Member States; and non-governmental organisations (NGOs), academic institutions and most Member States and international organisations. The different framings that are used by stakeholders with competing interests have consequences for the alcohol control policies that are considered and implemented at national and global level.

Alcohol is responsible for 5.1% of the global burden of disease from both non-communicable and infectious diseases, and 10% of the premature deaths among people aged 15-49 years.^1^ Alcohol use is also associated with secondary harms to those close to heavy drinkers, such as children^2^ and intimate partners,^3^ and contributes to wider societal harms due to alcohol-related violence, crime and road traffic incidents.^4-6^ These harms are greater among those who are more socio-economically deprived,^7,8^ rendering alcohol a key driver of social and health inequities. In order to address these issues, the World Health Organization (WHO) Global Strategy to Reduce the Harmful Use of Alcohol (GAS) was endorsed by the 63rd World Health Assembly in May 2010.^9^ The GAS aims to raise global awareness about alcohol harms; to strengthen the knowledge base on the determinants of alcohol harms and effective interventions; to increase technical support and Member State capacity; to strengthen partnerships and increase resource mobilization; and to improve systems of monitoring and surveillance.^9^ However, despite alcohol control efforts at national and global level, a 2019 WHO discussion paper on the implementation of the GAS concluded that the strategy did not result in a global decrease in alcohol consumption.^10^ According to data from the WHO *Global Status Report on Alcohol and Health*, the European Region was the only area where per capita alcohol consumption decreased between 2010 and 2018. On the other hand, certain regions of the world, most notably South-East Asia, saw an increase in alcohol consumption.^10^ The implementation of evidence-based alcohol policy was similarly more successful in high-income countries than in low- and middle-income countries.

 The discussion paper on the implementation of the GAS was led by the WHO and drew on data collection and consultations with Member States. Participation from other societal stakeholders was sought through a web-based consultation.^11^ The consultation was intended to give stakeholders the opportunity to share their perspectives on the implementation of the GAS and priority areas for future action. Consultations are used by governments and international organisations with the stated intention of giving affected parties an opportunity to provide information, evidence, and express their opinion about what and how decisions should be taken. In these ways, those taking part in consultations can contribute to the evidence and feedback gathering stage of the policy-making process.^12^ If well-conducted, consultations provide useful information and perspectives, and make decision-making processes more just and equitable by giving civil society access to debates that are usually only accessible to public bodies and lobbyists.

 However, consultations can also be used by vested interests to attempt to influence policy processes in ways that undermine public health.^13^ Analysis of public consultations can allow for the exploration of such strategies and provides insights into how a particular topic is conceptualised and/or contested by different stakeholders. This is particularly likely in the case of alcohol harms, where in Ralston and colleagues’ terms, there are well-documented ‘fault lines’ between commercial and other non-state actors.^14,15^ Other research documents the way in which industries, such as the tobacco and alcohol industries, manipulate the consultation process to disseminate industry framings, amplify their voice, and over-burden the process with substantial volumes of material.^16-19^ Identifying and analysing such framing is a precondition for developing counter frames that can challenge industry interpretations.

 In this paper, we analyse the responses to WHO’s consultation on the implementation of the GAS. Our analysis aims to assess how different stakeholders frame the problem of harmful use of alcohol, its causes and proposed solutions in their submissions, and how their framing is justified and sustained in order to advance a specific agenda.

## Methods

###  The Data

 Between October 24, and November 4, 2019, the WHO hosted a web-based consultation on the Discussion Paper *‘Implementation of the WHO global strategy to reduce the harmful use of alcohol during the first decade since its endorsement, and the way forward.’*^10^ The consultation was open to WHO Member States and governmental institutions, United Nations (UN) and other inter-governmental organisations (IGOs), non-governmental organisations (NGOs), academic institutions, and private sector entities (ie, trade associations and industry-funded NGOs, charities, and social aspects organisations [organisations that deliver the alcohol industry’s corporate social responsibility goals]). For this analysis, we obtained all 189 submissions from the WHO website,^11^ of which 161 were unique submissions (Table 1; see full list in Supplementary file 1). Five submissions written in French and four written in Spanish were translated by CR and MP and included in the analysis (Supplementary file 1). Attachments to the submissions that contained either the full response to the consultation or presented additional information written as part of an organisations’ response to the consultation were also analysed. Attachments that were not directly relevant to the consultation (eg, campaigns, evaluations, legal documents) were not included in the data set as the strategic use of such documents was deemed to be outside the scope of this framing analysis.

**Table 1:** Overview of Submissions to the Web-Based Consultation on the Implementation of the WHO GAS Since its Endorsement, and the Way Forward

**Stakeholder Group **	**Number of Submissions**	**Number of Unique Submissions**	**Submission Length (Words) **	**Number of Attachments **	**Number of Attachments Analysed** ^d^
Member States and governmental institutions	29	29	109-2058	5	0
UN system and other IGOs	4	4	166-684	2	1
Academic institutions	7	7	119-3487	4	0
NGOs	106	80^b^	72-7703	50	3
Private sector entities^a^	43	41^c^	322-3727	29	3
**Total**	189	161	-	90	7

Abbreviations: WHO, World Health Organization; GAS, Global Strategy to Reduce the Harmful Use of Alcohol; UN, United Nations; IGOs, inter-governmental organisations; NGOs, non-governmental organisations. ^a^ Trade associations and industry-funded NGOs, social aspects organisations and charities (of which one was originally submitted as an ‘NGO’). ^b^ The International Organisation of Good Templars’ (IOGT) response to the consultation was submitted 20 times in total by its member organisations and other organisations endorsing its response. The Global Alcohol Policy Alliance’s response was submitted seven times in total. The Alcohol Health Alliance and the Institute of Alcohol Studies submitted the same response. ^c^The Caribbean Breweries Association, Regional Beverage Alcohol Alliance, and Trinidad & Tobago Beverage Alcohol Alliance submitted the same response. ^d^Attachments presenting additional information written in response to the consultation, after removal of duplicates.

###  Theoretical Approach

 The consultation submissions were analysed using an inductive coding approach focusing on identifying the framing employed within the texts. A frame, or the act of framing, may be conceptualised as a principle of organisation, a way of comprehending and assigning subjective meaning to an event or issue, and in that way promoting a particular way of seeing the world while concealing or marginalising other ideas, explanations and viewpoints.^20,21^ Framing serves to promote particular ideas and perspectives and render them as established ‘objective’ truths, that is, as the ‘taken-for-granted’ way of conceptualising and perceiving social reality.^22^ While frames themselves are sometimes seen as static, pre-existing ‘objects,’^22,23^ the interactive process of framing (ie, constructing and promoting frames) is by definition dynamic and actor-led and thus appropriate for the analysis of policy processes.^21^ In the policy-making arena, actors use framing to set the agenda and influence both how problems are understood and what policy solutions are considered.^23^ As such, framing organises previous knowledge and values around a policy problem and serves to guide the action taken in response.^21^ Through framing, actors may seek to have their frames and ideas adopted as the ‘common sense’ understanding of an issue. What emerges from this dynamic process is based on what aspects of the issue are emphasised, how they are named, categorised and narrated by those involved, and the extent to which certain frames become ‘normalised.’ While this sense-making exercise is a necessary aspect of policy-making, it is also a political act. Hence, even in absence of institutionalised decision-making power, actors can exercise power over policy-making processes by shaping the dominant discourse around a policy issue.^24^ Identifying the dominant framing can give more insight into policy-making processes and forms the basis of this analysis. Similar approaches using framing analysis have previously been applied to public health issues,^14,23,25-28^ and the analysis of health-related consultation submissions.^15,29^ Due to the structured nature of the WHO consultation, the current analysis draws from Entman’s four framing functions: defining the problem, diagnosing causes, making moral judgements and suggesting remedies.^20^

###  Data Analysis

 In the first stage of the analysis, CR, MCvS and MP coded and discussed six submissions from different stakeholder groups to create an initial, non-exhaustive coding list to act as a guide to the subsequent coding. All submissions were then open coded independently by CR, MCvS and MP using Excel and NVivo 12. The 52 unique codes that emerged from the data were reviewed by CR to ensure coherence in coding between authors. Discrepancies were resolved through discussion. To minimise the extent to which the framing analysis would be influenced by previous framing studies of alcohol policy stakeholders,^14,25^ the coded data was anonymised by removing any reference to the submitting organisation. Frames were identified by CR, MCvS and MP by repeatedly reading the anonymised coded data. Coding was conducted using an ‘open’ approach, meaning it was guided by conceptual codes based on framing theory (eg, defining the problem, naming, categorising, narrating, assigning causation, proposing solutions)^20,21^ but also complemented by the identification of emergent codes as the data was analysed.^30^ This was an iterative process with several rounds of discussions amongst authors to reach consensus on framing functions and frames, and their interplay. Emergent frames were reviewed independently by a fourth researcher (ME). Frames were organised into higher order categories based on their framing function, a process previously identified to facilitate greater interpretation of the data, the interaction between different frames and the integration of theory.^23^ Once consensus was reached among all researchers through open discussion, individual stakeholders and stakeholder groups (ie, Member States, IGOs, NGOs, academic institutions and private sector entities) were linked to the identified policy framings. This was done with attention to both differences *between* and *within* stakeholder groups, thus recognising that these ‘groups’ are not necessarily homogeneous in terms of their use of framing.^31^

## Results

 Twelve frames were identified in the consultation submissions and grouped according to their function: (1) defining the problem, (2) assigning causation, (3) proposing solutions, or (4) justifying and persuading (Table 2, see Supplementary files 2-5 for an expanded version). These four framing functions are presented separately in this analysis but were often found to be interlinked. Certain framings of the ‘problem,’ such as that alcohol harm is an issue for a minority of ‘at risk’ individuals, similarly function to foreground and implicitly suggest that the solution must ‘target’ this ‘high-risk’ minority.

**Table 2:** Overview of the Framing That Emerged From the Submissions, and Their Categorisation Into Framing Functions

**Framing Functions**	**Frames**	**Selected Quotes**
Defining the problem	**The (harmful) use of alcohol ** Harmful use of alcohol, especially by individuals in at-risk groups or who engage in risky behaviours, is the problem Alcohol use (eg, per capita consumption) is the problem	“*Put more emphasis on harm indicators related to HED, Underage drinking, mortality and morbidity linked to harmful use of alcohol*” (CEEV, trade association). “*The term “harmful use” is misleading given the Global Burden of Disease Study suggested the level of consumption that minimizes health loss is 0”* (School of Public Health, The University of Hong Kong, academic institution).
	**Narrowing versus broadening the scope of the problem ** Negative consequences for individuals in at-risk groups or who engage in risky behaviours Negative health, social and economic consequences for wider society	*“With evidence (in Australia) that more consumers who drink are choosing to moderate their consumption, identifying cohorts where alcohol issues exist and addressing those risks should be the focus” *(DrinkWise, industry-funded charity). *“Alcohol consumption risks harm to the welfare not only of the drinker but also of others, and can impede sustainable social and economic development” *(Kettil Bruun Society for Social and Epidemiological Research on Alcohol, NGO).
Assigning causation	**Individual choice: **Alcohol misuse is caused by uninformed individual choices	“*For general consumers, the priority is to ensure that the consumers are best informed about the choices they make*” (Drinks Ireland, trade association).
	**Social norms: **Alcohol misuse is a deep-rooted social norm	“*The ultimate challenge, to cite the Discussion Paper, is that “Alcohol consumption is embedded in social norms and traditions,” which makes it a more complex and difficult behaviour to change*” (Drinkaware, industry-funded charity).
	**Under-regulation: **Alcohol misuse is caused by the widespread and underregulated availability, affordability and marketing of alcohol	“*Over the past decade, the world has not seen progress regarding alcohol prevention and control: neither in reducing alcohol consumption and related harm, nor in increasing alcohol policy best buy implementation*” (IOGT, NGO).
Proposing solutions	**Targeted interventions versus population-level regulation** Broad ‘menu’ of policy options targeted at at-risk individuals or risky behaviours Universal implementation of the three ‘best buys’ at population level	*“Heaviest drinkers, including heavy episodic drinkers, are the least sensitive to pricing policies. To be effective, a regulatory framework, which includes taxation, must be accompanied by interventions aimed specifically at harmful drinking” *(Brazilian Beer Trade Association, trade association). *“Over the last decade, WHO and health researchers confirmed the relevance of the so called ‘best buys’ to cost-effectively reduce and prevent alcohol related harm. Therefore, continuation of these policy directions should be envisaged”* (European Alcohol Policy Alliance, NGO).
	**Local versus global solutions** Local action specific to the national context (no ‘one size fits all’) Global action to address cross-border issues and industry interference	*“Where improvements have been made, a crucial element has been the recognition that harmful use of alcohol often requires tailored, local and culturally-sensitive measures*” (AssoBirra, trade association). *“We also see a great value in international cooperation in any form to address the cross-border issues of alcohol policy*” (Ministry of Social Affairs of Estonia, Member State).
	**Partnership versus freedom from industry interference** Involvement from a broad array of stakeholders, including industry The public sector should be independent from industry interference	*“Governments and international organisations need to work with industry and communities to develop evidence-based, targeted and effective initiatives to reduce harmful drinking”* (Australian Grape and Wine Inc., trade association). *“Countries need stronger support with technical capacity building regarding alcohol policy formulation, implementation, monitoring and safeguarding alcohol prevention and control efforts from the alcohol industry”* (IOGT, NGO).
Justifying and persuading	**Use of evidence: **To support or oppose problem definitions, causes and solutions	*“Duty increases have been evidenced to save lives and reduce harm (…)”* (Humankind Charity, NGO). “*The relation between Per Capita consumption or Prevalence and Harmful Consumption cannot be directly linked, more studies are required” *(Cerveceros Latinoamericanos, trade association).
	**Tobacco control analogies: **Alcohol control requires a similar approach to tobacco control	*“In line with what happens with tobacco, in article 5.3 of the FCTC, and in recognition of the inherent conflicts of interest that exist, there need to be strict rules prescribing the types of engagement permitted between states and international agencies on the one hand and economic operators/the liquor industry on the other”* (South African Medical Research Council, Member State).
	**Use of value systems** Neoliberal values: Individuals have the right to consume alcohol, and alcohol harms should be addressed by the private sector Human rights values: Alcohol and its widespread harms violate basic human right and should be regulated against	*“We need to recognise that encouraging responsible consumption will reduce alcohol related harm and the associated health and externality costs. History shows that prohibition drives illicit consumption, health related risk and increased abuse. We require a proportionate response”* (Australian Grape and Wine Inc., trade association). *“We support the inclusion of reference to relevant human rights and human rights instruments such as for example Article 12 of the International Covenant on Economic, Social and Cultural Rights* (…)” (McCabe Centre for Law & Cancer, NGO).

Abbreviations: CEEV, Comité Européen des Entreprises Vins; WHO, World Health Organization; NGO, non-governmental organisation; IOGT, International Organisation of Good Templars; HED, heavy episodic drinking; FCTC, Framework Convention on Tobacco Control.

###  Defining the Problem 

####  Defining the (Harmful) Use of Alcohol

 Two competing framings of the conceptualisation of the (harmful) use of alcohol were identified. The first perspective adopts a limited view, seeing alcohol consumption as an issue only when undertaken to what is referred to as ‘excessive’ consumption as opposed to an undefined ‘moderation’ – especially by individuals in at-risk groups (eg, minors, pregnant women) or those who engage in specific risky behaviours (eg, drink driving). This framing was adopted by all private sector entities, two Member States (Permanent Representation of Italy to the International Organizations and the United States) and the UN Conference on Trade and Development (IGO). Many of their submissions used framing that emphasised the ‘excessive’ use of alcohol to call for the WHO to continue its focus on the harmful use of alcohol and associated indicators exclusively, for example: *“indicators of HED [heavy episodic drinking] and alcohol related mortality and morbidity are much more relevant in measuring progress against the Global Alcohol Strategy than changes in per capita consumption”* (The UK alcoholic drinks trade associations, trade association). While additional indicators for alcohol harms are useful and already in use by WHO, many of these submissions appear to suggest that per capita alcohol consumption should not be emphasised at all, thus minimising its contribution to alcohol harm. Examples include the statements: “*The focus going forward should continue to be on tackling harmful drinking”* (The UK alcoholic drinks trade associations) and *“It is important that the focus remain on the harmful use of alcohol, not consumption per se” *(West Indies Rum & Sprits Producers Association, trade association).

 Another framing posits alcohol use in general (often defined as ‘per capita consumption’ or simply ‘alcohol consumption’) as the problem and appears to be based on the ample evidence that any level of consumption can be harmful. Most NGOs and academic institutions adopted this framing to call for a shift in language and focus, and suggested that replacing the term ‘harmful use of alcohol’ with ‘use of alcohol’ *“would alleviate confusion and support countries and people in understanding the degree of risk associated with alcohol use” *(NCD Alliance, NGO). However, the two identified framings of the problem definition are not necessarily mutually exclusive. Some NGOs, academic institutions and Member States emphasised both the risks associated with alcohol consumption in general and those of harmful consumption by specific vulnerable groups (eg, children and youth), who were believed to require additional protection. Unlike most industry submissions that limited the problem definition to ‘excessive’ use of alcohol, these submissions did not make a case for (exclusively) focusing on more harmful manifestations of alcohol use. They also adopted language that emphasised the need to protect vulnerable groups, rather than focusing on them or their behaviours as ‘the problem.’

####  Narrowing Versus Broadening the Scope of the Problem 

 While no organisation explicitly denied that alcohol causes significant harms in their submission, some stakeholders narrowed the focus of such harms to problematic individuals. This framing was mostly put forward by private sector entities, who also tended to focus exclusively on ‘harmful use of alcohol’ as opposed to consumption per se. These stakeholders tended to explicitly dismiss a wider population framing with the argument that *“identifying cohorts where alcohol issues exist and addressing those risks should be the focus”* (DrinkWise, Australian industry-funded charity) and that *“abstinence guidelines should therefore target […] dependent and problematic drinkers, not moderate drinkers”* (Educ’alcool, industry-funded charity, translated). Some private sector entities also referred to secondary harms compatible with this narrower focus on a sub-populations’ risky behaviours, such as foetal alcohol spectrum disorders resulting from drinking during pregnancy; or fatalities resulting from drink driving. For example, The Caribbean Breweries Association (trade association) acknowledged that *“[ i ]n 2015, the Dominican Republic had the highest estimated rate of fatalities from road accidents in the Americas,”* which it partly attributed to “harmful drinking” and drink driving.

 Conversely, the broader view that alcohol use has negative consequences for society as a whole was typically proposed by NGOs, academic institutions, Member States and IGOs. Their framing of the problem emphasised the importance of widening the scope of alcohol control to noncommunicable diseases as well as to alcohol’s *“impact on families and communities, its detrimental effects on productivity and economic growth, and its role in stressing and weakening already under-resourced health systems in low- and middle-income countries”* (Kettil Bruun Society for Social and Epidemiological Research on Alcohol, NGO).

###  Assigning Causation 

 The different ways in which stakeholders framed the problem of (harmful) use of alcohol appeared to be partnered with framings assigning different causes to the problem. Typically, these were individual choice, social norms and under-regulation.

####  Individual Choice

 According to the ‘individual choice’ framing, harms associated with alcohol are caused by a minority who do not drink ‘responsibly.’ None of the submissions included a definition of what was meant exactly with ‘responsible drinking.’ Harmful drinking choices were framed as being a consequence of lack of information and awareness about the risks associated with alcohol, emphasising *“the capacity to make positive changes, and therefore good health choices”* (Drinkaware, industry-funded charity). Another claimed reason for ‘irresponsible’ behaviour is the insufficient availability of alternative beverages with no or lower alcohol content. Such framing was most commonly adopted by private sector entities, as well as (among others) the Permanent Representation of Italy to the International Organizations, Latvia’s Ministry of Agriculture, and the Ministry of Social Affairs of Estonia (Member States).

####  Social Norms

 Alcohol (mis)use was also framed as being driven or influenced by social norms and traditions, which are suggested by some stakeholders to make it harder to elicit behaviour change. While social norms were often mentioned by private sector entities, this framing was also used by some Member States and NGOs to explain challenges in the implementation of the GAS. Arguments used in support of this framing include appeals to the history of alcoholic beverages, such as that “*[w] ine has been linked to human history since its inception and is part of the roots of the land, culture, tradition, history and pride of many countries” *(Vinos de Chile, trade association).

####  Under-regulation 

 The framing of alcohol harms as a problem caused by the widespread and under-regulated availability, affordability and marketing of alcoholic products was adopted by the majority of NGOs, academic institutions, Member States and IGOs. Policies to restrict availability, affordability and marketing have previously been described as the most effective and cost-effective ‘best buys.’^32^ Numerous submissions alluded to one or more best buys (sometimes using that term) and suggested that under-regulation was caused by a lack of political will and resources at the national level, as well as *“[t]he intrusion of alcohol industry in the global public health policy arena” *(Global Alcohol Policy Alliance, NGO) which has undermined *“governments’ ability to act on and progress effective regulations and policies”* (The Pacific Community, IGO), especially in low- and middle-income countries.

 Lack of resources as a reason for the limited national progress was also identified by private sector entities, which used it to highlight the economic benefits of industry involvement in alcohol control. However, most private sector entities and three Member States were critical of the concept of ‘commercial determinants of health’ (“strategies and approaches used by the private sector to promote products and choices that are detrimental to health” p. e895)^33^, which according to them *“risks portraying private sector engagement in a largely negative manner” *and “*negates the positive work of the private sector in activities that directly promote public health” *(The United States, Member State).

###  Proposing Solutions

 How stakeholders frame the problem and causes of alcohol-attributable harms has consequences for the (policy) solutions that they propose and who is held responsible for implementing them (eg, individuals, producers, governments or the WHO). The submissions to the consultation revealed three aspects of alcohol control that were framed differently by different stakeholders: the target population, the scale of policy solutions and the stakeholders that should be involved in alcohol control efforts.

####  Targeted Interventions Versus Population-Level Regulation

 In the submissions from private sector entities and five Member States (Permanent Representation of Italy to the International Organization, Latvia’s Ministry of Agriculture, the United States, Ministry of Health and Social Protection of Colombia and Guyana Mission), alcohol control was framed as requiring a targeted approach focused on at-risk individuals and risky behaviours. A targeted strategy in these submissions typically promoted a *“menu of policy options*,” including (industry-funded) education campaigns to promote ‘moderation,’ and responsible marketing practices. Many of the same stakeholders also applied this targeted approach to regulation by emphasising drink-driving legislation and legislation to prevent alcohol sale to minors instead of the best buys, which they argued would encourage illicit alcohol production and trade. Concern about illicit alcohol was similarly expressed by the UN Conference on Trade and Development (IGO), which referred to it as *“the worst form of harmful consumption of alcohol.”* Six NGO submissions (including those of various branches of the Green Crescent) also adopted a targeted approach based on education and awareness-raising interventions but did not encourage industry involvement.

 An alternative framing focused on universal, population-level regulation (ie, the implementation of one or more ‘best buys’) aimed at *“[s] trengthening rather than loosening the market controls on the availability, price and marketing of alcohol from the perspective of public health and the public interest”* (Ministry of Health Bulgaria, Member State) and *“promoting the principle that market regulation is fundamental to effective alcohol policy”* (TRAPS programme, The University of York, academic institution). This framing was adopted by the majority of NGOs, Member States, IGOs and academic institutions.

####  Local Versus Global Solutions

 Stakeholders that proposed targeted solutions to alcohol control often also favoured a localised approach specific to the national cultural, social and economic context, as opposed to what they sometimes called *“one size fits all”* regulation. The Mexican Chamber of the Tequila Industry (trade association) argued that due to different levels of commercialisation of alcohol in different countries “*we can assure that certain measures, especially those related to prices or alcohol taxation will not have any impact in the reduction of the harmful use of alcohol” *in certain countries.

 On the other hand, most NGOs, academic institutions, Member States and IGOs framed alcohol control as an increasingly global issue that requires international cooperation and global measures, including legal approaches to counteract challenges posed by the globalisation of the media environment, international trade agreements, and industry interference in policy-making. The most commonly proposed solution was a global legally binding regulatory framework on alcohol control similar to the Framework Convention on Tobacco Control (FCTC). This was suggested by 70 NGOs (66%), five academic institutions (71%), six Member States (20%) and one IGO (25%). Such a framework would among others “*counterbalance and constrain negative effects on alcohol policies of international trade and investment laws” *and “*constitute a strong symbolic statement, denormalising alcohol as a commodity and foodstuff” *(Australasian Professional Society on Alcohol and other Drugs, NGO).

####  Partnership Versus Freedom From Industry Interference 

 When it comes to suggesting who should be involved in alcohol control, one framing emphasised the importance of partnership with industry stakeholders. This framing was supported by all private sector entities, some more pro-industry Member States and IGOs (United States or America, Guyana Mission, Permanent Representation of Italy to the International Organizations, Ministry of Health and Social Protection of Colombia, and UN Conference on Trade and Development) and one NGO (International Federation of Medical Students’ Association). Most of these stakeholders promoted public-private partnerships, self-regulation (eg, marketing codes and voluntary labelling initiatives), co-regulation and industry corporate social responsibility activities. The partnership argument supports a *“whole of society approach”* as laid out in the WHO GAS and the UN Political Declaration on Non-communicable Diseases. Some stakeholder submissions favouring partnership with industry also dismissed:

 “(…) *the assumption that all collaboration with the private sector has a deep-set and unsurmountable conflict of interest, thus foregoing a previous evaluation of conditions, work and evidence. As has been proven above, this doesn’t have to be so”* (Fundación de Investigaciones Sociales A.C., social aspects organisation).

 On the other hand, most NGOs, academic institutions, Member States and IGOs supported freedom from alcohol industry interference and emphasised that, due to inherent conflict of interest, the public sector should exclude the alcohol industry from decision-making about alcohol control. These stakeholders also criticised the role given to the private sector in the WHO GAS for compromising the principle “*that all involved parties have the responsibility not to undermine implementation of policies to reduce harmful use of alcohol, and that public health should be given deference in relation to competing interests” *(The SPECTRUM Consortium, academic institution). Many stakeholders that used this framing suggested that the WHO is best equipped to provide countries with the financial and technical support so their alcohol policy development work has independence from the alcohol industry,* “primarily through enhancing the structure of the alcohol policy work to support regional offices and Member States; taking a stronger approach to industry interference; and developing guidelines for what engagement should occur at the national and global levels” *(Foundation for Alcohol Research and Education, NGO).

###  Justifying and Persuading 

 Stakeholders drew on evidence, comparisons between tobacco and alcohol control, and specific value systems to form justifying frames that support the adoption of certain problem and solution ideas and to oppose competing framing.

####  The Use of Evidence 

 Drawing upon government or industry data and peer-reviewed research evidence (or lack thereof) were the most common ways of justifying problem definitions, causes and proposed solutions in the submissions. However, not all statements that were claimed to be supported by evidence in the submissions are consistent with the current scientific consensus on alcohol harms and control. Statements that are unfounded or misleading given the current evidence base, such as that *“[o]ne fact is indisputable: moderate alcohol users have a longer life expectancy than abstainers for life” *(Educ’alcool, industry-funded charity, translated), were often made by private sector entities. However, some were also found in Member State submissions that used similar framing, for example the claim that *“while alcohol abuse produces serious implications for human health, moderate consumption does not imply particular risks, and can even produce positive effects” *(Permanent Representation of Italy to the International Organizations, Member State).

 Most of such statements contained claims that were either not supported with references or that misinterpreted the referenced studies. An example of the latter is the statement that *“alcohol consumption is not tied to physical availability of alcohol”* (Distilled Spirits Council of the United States, trade association), which is based on one study that found a mismatch between the concentration of alcohol availability and an increased likelihood of being a heavy drinker in Californian neighbourhoods, and does therefore not support the strong claim that “*reducing outlets and hours of sale will not reduce heavy drinking or total alcohol consumption.”* The same trade organisation wrote that *“studies clearly show that when taxes are increased, the consumption of illicit alcohol increases”* in a paragraph that questions the effectiveness of alcohol taxation (alongside that of the best buys more generally). This statement references a study concluding that “*making alcohol more expensive and less available, and banning alcohol advertising, are highly cost-effective strategies to reduce harm*” (p. 2234), which was omitted in the submission.^34^ Two private sector entities supported their arguments with references to peer-reviewed studies supported by the industry-funded International Center for Alcohol Policies (now the International Alliance for Responsible Drinking), of which one has been critiqued in a letter to the editor on the basis of “faulty methodology,” “misconstrued interpretations” and “conflict of interest.”^35^

####  Tobacco Control Analogies

 Comparison between tobacco control and alcohol control is another framing used by actors to justify advocating for specific policy measures. The NGOs, academic institutions, Member States and IGOs that used this framing argued that since the harm caused by alcohol is comparable to that caused by tobacco, especially when considering wider societal costs, alcohol should receive the same level of urgency and control. They also cited evidence that the tobacco and alcohol industries use similar approaches to undermine policy to further justify an approach similar to the FCTC to be applied to the alcohol industry (especially Article 5.3, which protects public health policies from vested interests).

####  The Use of Value Systems 

 Stakeholders appeared to draw on distinct value systems or world views to justify and legitimise their framing in the submissions. This included values emphasising individual responsibility and freedom, and the right to express preferences through consumer behaviour, which will loosely be referred to as neoliberal values (focusing on neoliberalism as an ideology rather than a political project).^36^ These values were drawn upon by private sector entities and some Member States to justify their framing of the problem of harmful use alcohol as a problem of individual responsibility with largely individual consequences, therefore requiring targeted interventions that give individuals the knowledge and tools to make informed choices. Stakeholders that used this framing argued that in the current GAS “*too much emphasis is put on prohibition and not enough on creating a stable and responsible environment” *(Belgian Brewers, trade association).

 In some submissions, the right to liberty and individual choice was also extended to economic actors, which were believed to have the right to operate without undue state intervention. Private sector entities argued that governments should encourage responsible practices by the private sector, such as self-regulation and the creation of beverages with low or no alcoholic content, instead of using regulations that *“create market distortions” *and *“may lead to unintended consequences, including growth in the unrecorded and illegal markets” *(Brazilian Beer Trade Association, trade association). At the same time, these stakeholders also stated that governments should be free to make decisions without interference from IGOs such as the WHO: *“For example, taxation is a sovereign right and any attempt to require a certain form or level of taxation is inappropriate”* (Spirits New Zealand, New Zealand Winegrowers and the Brewers Association of New Zealand, trade associations).

 Stakeholders that emphasised the broader harms of alcohol and the need for regulation and international tools often drew on human rights values. Harmful commodities such as alcohol and its harms to society were portrayed as undermining among others the right to the highest attainable standard of physical and mental health, as well as the realisation of the UN Sustainable Development Goals. As a consequence, many NGOs and academic institutions called for all people – and especially children and adolescents – to be protected from exposure to harmful alcohol consumption, marketing practices and other commercial influence, and alcohol-associated health and secondary harms. Protection of public health was seen as both a responsibility and right of national governments, and to a smaller extent the WHO:

*“[The right to health] outlines the state’s duty to protect people from an infringement of their right to health by third parties - including corporations. If products are being consumed in a manner hazardous to health, an obligation is placed on the state to intervene to protect the right to health. This requires nations to develop laws and policies at the domestic level that meet these minimum international standards” *(Alcohol Focus Scotland, NGO).

## Discussion

###  Main Findings and Relation to Previous Literature 

 This comprehensive analysis of consultation submissions on the implementation of the WHO GAS demonstrates the divergent framing adopted by different policy actors. Differences in framing were found between: private sector entities, five Member States (Permanent Representation of Italy to the International Organizations, United States of America, Guyana Mission, Ministry of Health and Social Protection of Colombia and Latvia’s Ministry of Agriculture) and one IGO (UN Conference on Trade and Development), who supported a targeted partnership approach to alcohol control focused on encouraging individual responsibility and behaviour change; and the remainder of the Member States and IGOs, as well as NGOs and academic institutions who predominantly promoted population-level regulation and global action.

 While the focus of this analysis was not to compare these two groups directly, our findings are largely consistent with previous research analysing the framing and arguments used by harmful commodity industries compared to those used by government and public health stakeholders.^15,28,37^ Such studies are however scarce in the field of alcohol control, where most research focuses exclusively on industry framing.^14,25,27,38^ By including submissions from a wide range of societal stakeholders, we identified strong support for the denormalization of alcohol industry involvement in public health policy-making among civil society and most Member States. Stakeholders thus went beyond the policy problems themselves, to reframe the identity and role of the alcohol industry within the global alcohol policy space. Our analysis also shows divergence in framing among stakeholders assigned to the same stakeholder ‘group,’ which is most notable among Member States. The United States of America (which has also been found to use industry-friendly framing in a 2017 consultation on the WHO draft tool to prevent and manage conflicts of interest in the development and implementation of nutrition programmes^15^) and the Permanent Representation of Italy to the International Organizations (representing Italy in various fora including the WHO, Human Rights Council, World Trade Organization and World Economic Forum) were the biggest outliers and consistently adopted framing that mirrored that identified among private actors.

 At the same time, the analysis of submissions by private sector entities confirms the consistency by which industry actors frame alcohol consumption and control over time and in different contexts.^14,19,38,39^ Examples include diverting attention towards individual responsibility as the main determinant of and solution to alcohol misuse (while directly opposing population-level measures), and positioning industry actors as important stakeholders in alcohol control.^40^ In addition, harmful commodity industries have a long history of challenging and casting doubt on scientific evidence of the harmful effects of their products or the effectiveness of policy measures, in order to subvert government regulation,^41,42^ for example through the use of neutral appearing charities or social aspects organisations to spread (mis)information in line with industry interests^25,43^ and funding research that supports industry interests^39,41,42^ – examples for both were found in the analysed submissions. The framing adopted by private sector entities seemed to ignore the evidence that environmental factors are key factors contributing to alcohol misuse^44,45^ and that population-level strategies are the most effective form of alcohol control^32^ (compared to, for example, mass education campaigns,^46^ industry self-regulation of alcohol marketing^47^ and corporate social responsibility activities^48,49^). For public health advocates, this highlights the importance of identifying and disseminating effective counter frames to pre-empt the now well-documented ‘alcohol industry playbook.’^14,39-41,43,48-50^ To create wide support for public health and human rights-based approaches around alcohol consumption, ‘fact-checking’ exercises are likely not enough. Instead, the focus may need to be on actively challenging misleading and harmful framings in a way that appeals to emotions and engages with the audience. There are many channels through which public health advocates can put forward their own framings of alcohol control, including public consultations, policy discussions and (mass) media. Social media in particular is often used by alcohol industry stakeholders and social aspects organisations to share messages that align with their preferred framing of alcohol harms and policy solutions. For public health advocates, this is an opportunity to help shape the dominant discourse around alcohol by introducing frames based on public health evidence and values. When it comes to the dissemination of such framing, lessons can be learned from public health countermarketing campaigns, which are most common in the field of tobacco control. Such strategies aim “to reduce the demand for unhealthy products by exposing the motives of their producers and portraying their marketing activities as outside the boundaries of civilized corporate behaviour” (p. 120).^51^ Key framing could focus on adverse health and social consequences of alcohol consumption, exposing industry manipulation of consumers, policy-makers and the scientific process, their targeting of vulnerable populations, the environmental impact of alcohol production and human rights violations by the industry.^52,53^

 However, it is important to avoid the narrowly-focused narrative that a simple trade-off between local, individual-level action or global action at population-level exists. Rather these should be considered to be complementary strategies which are necessary to comprehensively address alcohol consumption (something that was already acknowledged in the submissions of some NGOs and academic institutions).

 In this context, it is also important to consider that framing is inherently political.^21^ By using framing analysis to understand the value systems or world views that underpin particular framings of the problem of alcohol harms, its causations and proposed solutions, we have tried to shed light on how discourse can be used in alcohol policy-making. Policy-making is a political process, and stakeholders use power and discourse to shape (or frame) evidence and argue for their policy preferences.^21,23,24^ For example, policies based principally on values of individual and corporate freedom and responsibility, as promoted by private sector entities, are likely to serve commercial interests over public health interests, and maintain power imbalances. This links with the wider academic debate about whether the ‘neoliberal’ policy paradigm that has become dominant since the 1980s enables increasingly powerful commercial interests to influence public policy while creating barriers for states to implement evidence-based public health measures.^36^ The prioritisation of economic outcomes alongside the view that economic growth will automatically lead to improvement in social outcomes, which is reflected in national policy-making and international trade agreements, is believed to lie at the root of this,^54^ creating conflict between governments’ economic and public health objectives and increasing the global supply of unhealthy commodities, especially in low- and middle-income countries.^55-57^

###  Implications and Wider Considerations 

 A legally binding treaty on alcohol control as proposed by numerous stakeholders in the consultation would offer legitimate means for Member States to prioritise the implementation of alcohol control measures in the face of pressure from industry and international trade rules. Yet, it should not be framed as the be-all and end-all solution to the harms caused by alcohol. Even the FCTC, the strongest instrument to control harmful commodities to date, has not been able to forge the necessary action by Member States to meet all the Treaty obligations (implementation rate ranged between 15% and 88% across Articles in 2016).^56,58,59^ Problems that were identified in the consultation submissions on the implementation of the GAS, including sustained industry interference and a lack of sufficient capacity and financial support, remain relevant for tobacco control.^56,59^ This suggests that public health advocates must remain vigilant to the framing and tactics of the alcohol industry even if it were to be formally excluded from deliberations and decision-making; and that increased capacity-building and resource mobilisation at the national level are central to the success of an international treaty like the FCTC. While there was very strong support for a ‘Framework Convention on Alcohol Control’ within the consultation, there was less recognition of the structures needed to translate such a treaty to actual change at the national level.

 The WHO has an important position in determining the future direction of global alcohol control (including the implementation of a binding treaty) given its normative role in global discourse and practices. How the WHO weighs upthe opinions and policy suggestions of different stakeholder groups that took part in the consultation on the WHO GAS is therefore important to understand. Not much detail of this is available on the WHO website, which mentions that *“[t]he comments received will be taken into consideration in the process of developing [a report to the 72nd World Health Assembly by the WHO Secretariat] and may serve as an input for informal consultation with Member States on the discussion paper.”* Earlier analyses indicate that WHO policy-making processes are relatively resistant to industry interference,^13,60^ also specifically at the consultation stage.^13^ However, it does become evident that the consultation hosted by the WHO in response to their discussion paper on the implementation of the GAS contains misleading statements, such as those casting doubt on the unhealthful effects of alcohol or potential solutions. It is therefore essential for the WHO to ensure that the decision-making processes which underlie consultations are objective, evidence-based and aligned with explicit values of improving the health of populations. At the same time, the WHO also has an important role to play in countering misinformation that could harm health. The submissions to the 2019 consultation on the implementation of GAS, some of which contain misinformation and misleading framing, are publicly available on the WHO website, which could lead to the inadvertent amplification of misinformation. We therefore welcome the disclaimer that has been added to the submissions of a more recent consultation on WHO’s ‘Working document for development of an action plan to strengthen implementation of the GAS.’^61^ This disclaimer makes clear that *“[t]he World Health Organization does not warrant that the information contained in this document is complete and correct.” *In addition to this, the WHO could consider including rebuttals or ‘tags’ when publishing submission to public consultations. This has been suggested as a way of combatting misinformation in other contexts.^62^

 There is a need for further analysis of the role of public consultations and the way in which they do or do not address the inherent power and resource imbalances that may exist between policy actors taking part, and the weight afforded to different agents. As Marks discusses in his in-depth analysis of partnerships with industry and industry’s role in policy-making, there needs to be more critical reflection on the implications of including industry in the policy-making process, as such practices clearly create channels for industry influence and misinformation.^63^ Marks also argues for greater recognition that within public consultations and other decision-making process, the views and interests of certain actors, such as the public, can be afforded greater weight than those of others who hold vested commercial interests in the outcome of specific policy decisions.^63^ This could also take into account areas of competence, and the recognition that alcohol producers are different from other stakeholder groups in the sense that they do not experience, pay for or study alcohol harms and policies to mitigate it. Considering the known strategies that the alcohol industry uses in an attempt to dominate the discourse around alcohol control and undermine public health efforts to address alcohol harms, the WHO should consider the call from NGOs, academic institutions and Member States to terminate its ‘dialogue’ with the alcohol industry, as is the case with the tobacco industry, and to create more meaningful means of participation for civil society. That being said, public health advocates can continue to strengthen their capacity to counter industry frames and develop their own persuasive evidence-based narratives and framing, regardless of whether industry takes part in WHO consultations.

###  Strengths and Limitations 

 There are various frameworks to analyse the political activities used by harmful commodity industries to influence policy-making, as well as various studies on the use of framing by alcohol industry actors.^14,25,26^ We adopted an inductive and iterative approach which allowed for the emergence and identification of framing from the data. Framing is a subjective practice rooted in social constructivism, which means that frames will be interpreted differently by different people. The framings that are presented above represent what was identified by four independent analyses of the data and finalised through discussion to reach consensus. A limitation of framing analysis applied to textual data like consultation submissions is that it cannot discern what motivations and intentions underlie the adoption of a particular framing. In the same way, framing analysis cannot predict how those assessing the worth of those submissions will respond to the frames and arguments adopted. Instead, we have sought to put our results in context using previous studies with different and complementary methodologies. Future research should address the implications of public consultations and other means to increase participation in deliberations about alcohol control organised by the WHO, to analyse the role of consultations in WHO’s final outputs and the influence of different stakeholders in the process.

## Conclusion

 Alcohol control is a contested policy field in which different stakeholders use different framing as part of their effort to set the agenda and influence what policy solutions are considered and adopted. Framing analysis is an important tool in policy analysis as it allows for the identification of the underlying assumptions and values of different policy options, and assists in their assessment. This analysis of submissions to a WHO consultation on the GAS identified two divergent framings: one that focuses on excessive alcohol use on an individual level and one that focuses on alcohol consumption as a collective, population-wide problem. The divergent framings have consequences for the strategies that are proposed for reducing alcohol harms. There is a need for greater consideration of the interests that are served by these different framings and how closely they align with WHO’s public health and health equity ambitions. Understanding this is a precondition for countering misleading frames. If improving the health of populations is to be made central in future global tools and strategies to prevent alcohol-related harms, WHO must carefully consider how it can ensure that engagement with industry in any form does not undermine public health goals, including during the process of formal consultations.

## Acknowledgements

 The authors would like to thank Dr. Mélissa Mialon, Dr. Ben Hawkins, and Dr. Nason Maani for their valuable comments on the manuscript.

## Ethical issues

 This study made use of publicly available secondary data. Ethics approval was not required for this study.

## Competing interests

 Authors declare that they have no competing interests.

## Authors’ contributions

 CR, MCvS, and MP designed the study and performed the data analysis. ME reviewed the data and validated the results. CR drafted the manuscript, with support from MCvS, MP, and ME.

## Funding

 No funding was received for this study. CR is funded by a National Institute for Health Research (NIHR) School for Public Health Research (SPHR) Pre-doctoral Fellowship. MCvS is funded by a NIHR Doctoral Fellowship (Ref NIHR300156). The views expressed are those of the authors and not necessarily those of the NIHR or the Department of Health and Social Care. MP is a member of the SPECTRUM Consortium, which is funded by the UK Prevention Research Partnership, an initiative funded by UK Research and Innovation Councils, the Department of Health and Social Care (England) and the UK devolved administrations, and leading health research charities.

## Supplementary files


Supplementary file 1. Submitting Stakeholders.

Supplementary file 2. Identified Framing Defining the Problem.

Supplementary file 3. Identified Framing Assigning Causation.

Supplementary file 4. Identified Framing Proposing Solutions.

Supplementary file 5. Identified Framing Justifying and Persuading.
