# Supplementary material for: A Framing Analysis of Consultation Submissions on the WHO Global Strategy to Reduce the Harmful Use of Alcohol: Values and Interests
Source: Int J Health Policy Manag. 2021 Jun 26;11(8):1550–61. doi: 10.34172/ijhpm.2021.68 (PMC9808336; doi:10.34172/ijhpm.2021.68)
Supplement: Supplementary file 1 — Submitting Stakeholders. [file ijhpm-11-1550-s001.pdf]

Article title: A Framing Analysis of Consultation Submissions on the WHO Global Strategy to Reduce the Harmful Use of Alcohol: Values and Interests

Journal name: International Journal of Health Policy and Management (IJHPM)

Authors' information: Chiara Rinaldi*[1], May CI van Schalkwyk[1], Matt Egan[2], Mark Petticrew[2]

[1]Department of Health Services Research and Policy, London School of Hygiene and Tropical Medicine, London, UK.

[2]Department of Public Health, Environments and Society, London School of Hygiene and Tropical Medicine, London, UK.

(*corresponding author: chiara.rinaldi@lshtm.ac.uk)

Supplementary file 1. Submitting Stakeholders

Table S1: Stakeholders that submitted a response to the web-based consultation on 'The implementation of the WHO global strategy to reduce the harmful use of alcohol since its endorsement, and the way forward'.

Stakeholder group	Organisations
Member States and governmental institutions	FPS Public health, Food chain safety and Environment, Belgium<br>NCPHA, MoH Bulgaria<br>Ministerio de salud y proteccion social de Colombia[a]<br>Cook Islands Ministry of Health<br>Instituto sobre Alcoholismo y Fármacodependencia (IAFA), Costa Rica[a]<br>Ministerio de Salud Pública de la República de Cuba[a]<br>Ministry of Health of the Czech Republic<br>Office of the Government of the Czech Republic, Drug Policy Department<br>The National Institute of Public Health, Czech Republic<br>Ministry of Social Affairs of Estonia<br>Permanent Mission of Georgia to the United Nations Office in Geneva and other international organizations<br>Guyana Mission<br>Directorate of Health, Iceland<br>Department of Health, Ireland<br>Permanent Representation of Italy to the International Organizations, Geneva<br>Ministry of agriculture, Latvia<br>Ministry of Health of the Republic of Latvia<br>Centre for Diseases Prevention and Control, Latvia<br>Ministry of Health, National Commission Against Addictions, Mexico<br>Ministry of Health Mozambique<br>Ministry of Health, Welfare and Sport, The Netherlands<br>Ministry of Health, New Zealand<br>The State Agency for Prevention of Alcohol Related Problems, Poland<br>Ministry of Health, Republic of Slovenia<br>South African Medical Research Council<br>Spanish Ministry of Health, Consumer Affairs and Welfare<br>Federal Office for Public Health, Switzerland[b]<br>Ministry of Public Health, Thailand

	United States of America
UN system and other international organisations	European Centre Social Welfare Policy and Research<br>UNDP<br>United Nations Conference on Trade and Development<br>The Pacific Community (SPC) (on behalf of Pacific Island Countries and Territories)
Academic institutions	Mental Health Institute and WHO Collaborating Center for Psychosocial Factor, Drug Abuse and Health, Central South University<br>School of Public Health, LKS Faculty of Medicine, The University of Hong Kong<br>SHORE Research Centre<br>Community Action on Youth and Drugs National Coordination Team, Massey University<br>MRC/CSO Social and Public Health Sciences Unit, University of Glasgow<br>TRAPS (Transformative Research on Alcohol Policy and Science programme at the University of York)<br>SPECTRUM (Shaping Public hEalth poliCies To Reduce IneqUalities and harM)
Non-governmental organisations (NGOs)	Abstinentenverband des Kantons Zürich<br>AESKAN<br>Afghanistan green crescent organization (AGCO)<br>Alcohol & Drug Information Centre (ADIC), India<br>Alcohol Action Ireland<br>Alcohol Action New Zealand<br>Alcohol and Drug Information Centre (ADIC)<br>Alcohol Focus Scotland<br>Alcohol Health Alliance<br>Alcohol Policy Alliance Gambia<br>Alcolisti Anonimi Italia<br>Amardeep India<br>APABurkina<br>Asia Pacific Alcohol Policy Alliance<br>Association for Promoting Social Action (APSA)<br>Association of Advocates against Alcohol Harm in Nigeria<br>Australasian Professional Society on Alcohol and other Drugs (APSAD)<br>Balance, the North East Alcohol Office<br>Canadian Centre for Substance use and Addiction (CCSA)<br>Cancer Society<br>Center for youth education<br>Centre for Alcohol Studies, Thai Health Promotion Foundation<br>Croissant Vert Nigerien (CVN)[b]<br>Cruz Azul no Brasil<br>EHYT Finnish Association for Substance Abuse Prevention<br>European Alcohol Policy Alliance<br>European Mutual help Network for Alcohol related problems (EMNA)<br>Fondacioni YESILAY<br>FORUT<br>Foundation for Alcohol Research and Education<br>Foundation for Innovative Social Development (FISD)<br>Global Alcohol Policy Alliance<br>Green crescent of Congo est[b]<br>Green Crescent Society, Turkey<br>Green Crescent South Africa<br>Green Crescent Zimbabwe<br>Green Crescents Kazakhstan<br>Green Moon

	Hāpai Te Hauora Tapui Limited<br>HealthBridge Foundation of Canada, Vietnam Office<br>Healthy India Alliance<br>Hong Kong Alliance for Advocacy Against Alcohol<br>Hope and Beyond<br>HRIDAY<br>Humankind Charity<br>Institute for Research and Development "Utrip"<br>Institute of Alcohol Studies<br>International Blue Cross<br>International Federation of Medical Students' Association (IFMSA)<br>International Youth Health Organizations<br>IOGT Gambia<br>IOGT Germany<br>IOGT Guinea-Bissau<br>IOGT Iceland<br>IOGT International<br>IOGT Norway<br>IOGT Switzerland<br>IOGT-NTO<br>IOGT-VN<br>Italian Society on Alcohol (SIA)<br>Junis<br>Juvente<br>Juvente Switzerland<br>Kettil Bruun Society for Social and Epidemiological Research on Alcohol<br>Liberia Alcohol Policy Alliance<br>Lithuanian Tobacco and Alcohol Control Coalition<br>McCabe Centre for Law & Cancer<br>Moroccan Green Crescent<br>Movendi slovakia<br>Núll Prósent Hreyfingin<br>Nada India Foundation<br>National Alliance for Action on Alcohol<br>NCD Alliance<br>Newcastle Coalition inner city resident groups, small businesses and concerned citizens<br>NGO “Zeleni krst - Zeleni polumjesec”, Serbia<br>Nigeria Alcohol Prevention Youth Initiative<br>Nordic Alcohol and Drug Policy Network (NordAN)<br>People Center for Development and Peace<br>Pioneer Total Abstinence Association<br>Public Union against Bad Habits<br>Recovery and Humanitarian Action Management Agency (RAHAMA)<br>RECOVERY, z.s.<br>Research and Training Center for Community Development (RTCCD)- The coordination organization of the Vienam Non-Communicable Diseases Control and Prevention Alliance (NCDs-VN)<br>Scottish Health Action on Alcohol Problems - SHAAP<br>Senegalese Alcohol Policy Alliance (SenAPA)<br>Serenity Harm Reduction Programme Zambia (SHARPZ)<br>Sierra Leone Alcohol Policy Alliance (SLAPA)<br>Southern African Alcohol Policy Alliance<br>Sri Lanka Medical Association<br>Stopdrink Network

	Students Campaign Against Drugs<br>Swedish cancer society<br>Tanzania Media Women's Association (TAMWA)<br>Tanzania Network Against Alcohol Abuse (TAAnet)<br>The Wellbeing Initiative<br>Trimbos Institute<br>Udruzenje Gradana Zeleni Polumjesec U Bih<br>Uganda Youth Development Link<br>UNF<br>United States Alcohol Policy Alliance (U.S.APA)<br>Value Health Africa<br>Vision for Alternative Development<br>WAAPA-Benin/ Secrétariat (Initiative pour l'Education et le Contrôle du Tabagisme)<br>West African Alcohol Policy Alliance (WAAPA)<br>World Federation Against Drugs<br>Youth against Alcoholism and Drug Dependency (YADD)
Private sector entities	ISWAI International Spirits & Wine Association of India<br>Alcohol Awareness Foundation Ireland (trading as Drinkaware)<br>Alcohol Beverages Australia<br>Asociación Dominicana de Productores de Ron (ADOPRON)<br>AssoBirra<br>Association for Alcohol Responsibility and Education (aware.org)<br>Association of Alcohol Manufacturers and Importers<br>Australian Grape and Wine Inc. Australian Grape & Wine)<br>Beer Canada<br>Beer Institute<br>Belgian Brewers<br>Brazilian Beer Trade Association (SINDICERV)<br>Bundesverband der Deutschen Spirituosen-Industrie und -Importeure e.V. (BSI)/Federal Association of the German Spirits Industry and Importers (BSI)<br>Caribbean Breweries Association (CBA)<br>CEEV, Comité européen des entreprises vins<br>Cerveceros de España<br>Cerveceros Latinoamericanos<br>CTA – Confederation of Business Associations of Mozambique<br>Distilled Spirits Council of the United Sates<br>Drinks Ireland<br>DrinkWise<br>Educ'alcool [b c]<br>Fédération des Exportateurs de Vins et Spiritueux de France (FEVS)[b]<br>FEDERACIÓN ESPAÑOLA DEL VINO (FEV)[a]<br>FIVS<br>Fundación de Investigaciones Sociales A.C. (Foundation of Social Research)<br>International Alliance for Responsible Drinking (IARD)<br>Japan Spirits & Liqueurs Makers Association (JSLMA)<br>México's National Chamber of Beer and Malt<br>Mexican Chamber of the Tequila Industry<br>Regional Beverage Alcohol Alliance (RBAA)<br>Representantes-Importadores de Vinos y Licores Asociados (RIVLAS)<br>South African Liquor Brand owners Association<br>Spirits New Zealand, New Zealand Winegrowers and the Brewers Association of New Zealand<br>spiritsEUROPE

	STIVA (Foundation for responsible alcohol consumption)<br>The Brewers of Europe<br>The UK alcoholic drinks trade associations: British Beer & Pub Association, National Association of Cider Makers, Scotch Whisky Association and Wine and Spirit Trade Association<br>Trinidad & Tobago Beverage Alcohol Alliance (TTBAA)<br>Vinos de Chile<br>West Indies Rum & Spirits Producers Association (WIRSPA)<br>World Spirits Alliance<br>Worldwide Brewing Alliance

[a] Submissions written in Spanish that were translated for this analysis.

[b] Submissions written in French that were translated for this analysis.

[c] While Educ'alcool originally submitted their response to the consultation in the 'NGOs' category, it was considered a 'private sector entity' in this analysis because of its ties with the alcohol industry, which provides part of its funding. This categorisation creates greater consistency in the data, given that other industry-funded NGOs, social aspect organisations and charities were already categorised as 'private sector entities' on the WHO website.
