# Supplementary material for: A Framing Analysis of Consultation Submissions on the WHO Global Strategy to Reduce the Harmful Use of Alcohol: Values and Interests
Source: Int J Health Policy Manag. 2021 Jun 26;11(8):1550–61. doi: 10.34172/ijhpm.2021.68 (PMC9808336; doi:10.34172/ijhpm.2021.68)
Supplement: Supplementary file 2 — Identified Framing Defining the Problem. [file ijhpm-11-1550-s002.pdf]

Article title: A Framing Analysis of Consultation Submissions on the WHO Global Strategy to Reduce the Harmful Use of Alcohol: Values and Interests

Journal name: International Journal of Health Policy and Management (IJHPM)

Authors' information: Chiara Rinaldi*[1], May CI van Schalkwyk[1], Matt Egan[2], Mark Petticrew[2]

1. Department of Health Services Research and Policy, London School of Hygiene and Tropical Medicine, London, United Kingdom (*corresponding author: Chiara.Rinaldi@lshtm.ac.uk
2. Department of Public Health, Environments and Society, London School of Hygiene & Tropical Medicine, London, United Kingdom

Supplementary file 2. Identified Framing Defining 'the Problem'

Table S2. Expanded table of the identified frames, submitting stakeholders and exemplifying quotes.

Frames		Quotes	Stakeholders
**Defining the (harmful) use of alcohol**	Harmful use of alcohol, especially by individuals in at-risk groups or who engage in risky behaviours, is the problem	"Put more emphasis on harm indicators related to HED, Underage drinking, mortality and morbidity linked to harmful use of alcohol instead of focusing only on national average pro capita consumption." (CEEV, trade association)<br><br>"A recent study looking at British consumption suggests that average alcohol consumption is not evenly distributed across all consumption levels and that drops in the population average may mask increase among heavy drinkers. The indicators of HED and alcohol related mortality and morbidity are much more relevant in measuring progress against the Global Alcohol Strategy than changes in per capita consumption. The focus going forward should continue to be on	**Member States and governmental institutions**<br>Permanent Representation of Italy to the International Organizations<br>United States of America<br>**UN system and other international organisations (IGOs)**<br>United Nations Conference on Trade and Development<br>**Private sector entities**<br>Alcohol Awareness Foundation Ireland (trading as Drinkaware)<br>Alcohol Beverages Australia<br>Asociación Dominicana de Productores de Ron (ADOPRON)<br>AssoBirra<br>Association for Alcohol Responsibility and Education (aware.org)<br>Association of Alcohol Manufacturers and Importers<br>Australian Grape and Wine Inc. Australian Grape & Wine)<br>Beer Canada<br>Beer Institute<br>Belgian Brewers<br>Brazilian Beer Trade Association (SINDICERV)<br>Bundesverband der Deutschen Spirituosen-Industrie und -Importeure e.V. (BSI)/Federal Association of the German Spirits Industry and Importers (BSI)<br>Caribbean Breweries Association (CBA)<br>CEEV, Comité européen des entreprises vins<br>Cerveceros de España<br>Cerveceros Latinoamericanos

		tackling harmful drinking." (The UK alcoholic drinks trade associations, trade association)	CTA – Confederation of Business Associations of Mozambique<br>Distilled Spirits Council of the United Sates<br>Drinks Ireland<br>DrinkWise<br>Educ'alcoola<br>FEDERACIÓN ESPAÑOLA DEL VINO (FEV)<br>Fédération des Exportateurs de Vins et Spiritueux de France (FEVS)<br>FIVS<br>Fundación de Investigaciones Sociales A.C. (Foundation of Social Research)<br>International Alliance for Responsible Drinking (IARD)<br>ISWAI International Spirits & Wine Association of India<br>Japan Spirits & Liqueurs Makers Association (JSLMA)<br>Mexican Chamber of the Tequila Industry<br>México's National Chamber of Beer and Malt<br>Regional Beverage Alcohol Alliance (RBAA)<br>Representantes-Importadores de Vinos y Licores Asociados (RIVLAS)<br>South African Liquor Brand owners Association<br>Spirits New Zealand, New Zealand Winegrowers and the Brewers Association of New Zealand<br>spiritsEUROPE<br>STIVA (Foundation for responsible alcohol consumption)<br>The Brewers of Europe<br>The UK alcoholic drinks trade associations: British Beer & Pub Association, National Association of Cider Makers, Scotch Whisky Association and Wine and Spirit Trade Association<br>Trinidad & Tobago Beverage Alcohol Alliance (TTBAA)<br>Vinos de Chile<br>West Indies Rum & Spirits Producers Association (WIRSPA)<br>World Spirits Alliance<br>Worldwide Brewing Alliance
	Alcohol use (i.e. per capita consumption) is the problem	"The term "harmful use" is misleading given the Global Burden of Disease Study suggested the level of consumption that minimizes health loss is 0." (School of Public Health, The University of Hong Kong, academic institution)	**Member States and governmental institutions**<br>Centre for Diseases Prevention and Control, Latvia<br>Cook Islands Ministry of Health<br>Federal Office for Public Health, Switzerland<br>Instituto sobre Alcoholismo y Fármacodependencia (IAFA), Costa Rica<br>Ministry of agriculture, Latvia<br>Ministry of Health of the Republic of Latvia<br>Ministry of Health, National Commission Against Addictions, Mexico<br>Ministry of Health, New Zealand

		"For example, the concept of "harmful use of alcohol" is highly problematic. It's a term that is confusing and supports the narrative and myths of the alcohol industry. The term disguises both the real magnitude of the alcohol burden (see above) as well as the motives of the alcohol industry for the public and policy makers. (…) But nearly ten years after the adoption of the WHO GAS it is now important to revisit, rethink and reframe - to allow WHO and other UN partners to communicate more effectively, clearly and compellingly about alcohol harm and the alcohol policy solutions." (IOGT, NGO)	Ministry of Health, Republic of Slovenia<br>Ministry of Health, Welfare and Sport, The Netherlands<br>Ministry of Public Health, Thailand<br>Ministry of Social Affairs of Estonia<br>NCPHA, MoH Bulgaria<br>Permanent Mission of Georgia to the United Nations Office in Geneva and other international organizations<br>South African Medical Research Council<br>Spanish Ministry of Health, Consumer Affairs and Welfare<br>The National Institute of Public Health, Czech Republic<br>**UN system and other IGOs**<br>European Centre Social Welfare Policy and Research<br>The Pacific Community (SPC) (on behalf of Pacific Island Countries and Territories)<br>UNDP<br>**Academic institutions**<br>Community Action on Youth and Drugs National Coordination Team, Massey University<br>MRC/CSO Social and Public Health Sciences Unit, University of Glasgow<br>School of Public Health, LKS Faculty of Medicine, The University of Hong Kong<br>SHORE Research Centre<br>SPECTRUM (Shaping Public hEalth poliCies To Reduce IneqUalities and harM)<br>TRAPS (Transformative Research on Alcohol Policy and Science programme at the University of York)<br>**Non-governmental organisations (NGOs)**<br>Abstinentenverband des Kantons Zürich<br>AESKAN<br>AFGHANISTAN GREEN CRESCENT ORGANIZAION (AGCO)<br>Alcohol & Drug Information Centre (ADIC), India<br>Alcohol Action Ireland<br>Alcohol Action New Zealand<br>Alcohol and Drug Information Centre (ADIC)<br>Alcohol Focus Scotland<br>Alcohol Health Alliance<br>Amardeep India<br>APABurkina<br>Asia Pacific Alcohol Policy Alliance<br>Association for Promoting Social Action (APSA)<br>Australasian Professional Society on Alcohol and other Drugs (APSAD)

			Canadian Centre for Substance use and Addiction (CCSA)<br>Cancer Society<br>Center for youth education<br>Centre for Alcohol Studies, Thai Health Promotion Foundation<br>Cruz Azul no Brasil<br>EHYT Finnish Association for Substance Abuse Prevention<br>European Alcohol Policy Alliance<br>European Mutual help Network for Alcohol related problems (EMNA)<br>Fondacioni YESILAY<br>FORUT<br>Foundation for Alcohol Research and Education<br>Foundation for Innovative Social Development (FISD)<br>Global Alcohol Policy Alliance<br>Green crescent of Congo est<br>Green Crescent Society, Turkey<br>Green Crescents Kazakhstan<br>Hāpai Te Hauora Tapui Limited<br>HealthBridge Foundation of Canada, Vietnam Office<br>Healthy India Alliance<br>Hong Kong Alliance for Advocacy Against Alcohol<br>Hope and Beyond<br>HRIDAY<br>Institute for Research and Development "Utrip"<br>Institute of Alcohol Studies<br>International Blue Cross<br>International Federation of Medical Students' Association (IFMSA)<br>International Youth Health Organizations<br>IOGT Gambia<br>IOGT Germany<br>IOGT Guinea-Bissau<br>IOGT Iceland<br>IOGT International<br>IOGT Norway<br>IOGT Switzerland<br>IOGT-NTO<br>Italian Society on Alcohol (SIA)<br>Junis<br>Juvente<br>Juvente Switzerland

			Kettil Bruun Society for Social and Epidemiological Research on Alcohol<br>Liberia Alcohol Policy Alliance<br>Lithuanian Tobacco and Alcohol Control Coalition<br>Moroccan Green Crescent<br>movendi slovakia<br>Nada India Foundation<br>National Alliance for Action on Alcohol<br>NCD Alliance<br>Newcastle Coalition inner city resident groups, small businesses and concerned citizens<br>Nigeria Alcohol Prevention Youth Initiative<br>Nordic Alcohol and Drug Policy Network (NordAN)<br>Núll Prósent Hreyfingin<br>Recovery and Humanitarian Action Management Agency (RAHAMA)<br>Research and Training Center for Community Development (RTCCD)- The coordination organization of the Vienam Non-Communicable Diseases Control and Prevention Alliance (NCDs-VN)<br>Scottish Health Action on Alcohol Problems - SHAAP<br>Senegalese Alcohol Policy Alliance (SenAPA)<br>Sierra Leone Alcohol Policy Alliance (SLAPA)<br>Southern African Alcohol Policy Alliance<br>Sri Lanka Medical Association<br>Stopdrink Network<br>Students Campaign Against Drugs<br>The Wellbeing Initiative<br>Trimbos Institute<br>United States Alcohol Policy Alliance (U.S.APA)<br>Vision for Alternative Development<br>WAAPA-Benin/ Secrétariat (Initiative pour l'Education et le Contrôle du Tabagisme)<br>West African Alcohol Policy Alliance (WAAPA)<br>World Federation Against Drugs<br>Youth against Alcoholism and Drug Dependency (YADD)
**Narrowing versus broadening the scope of the problem**	Negative consequences for individuals in at-risk groups or	"Alcohol related harms are not experienced uniformly across the population. With evidence (in Australia) that more consumers who drink are choosing to moderate their	**Member States and governmental institutions**<br>Ministerio de salud y proteccion social de Colombia<br>Permanent Mission of Georgia to the United Nations Office in Geneva and other international organizations<br>The State Agency for Prevention of Alcohol Related Problems, Poland<br>**Private sector entities**

	who engage in risky behaviours	consumption, identifying cohorts where alcohol issues exist and addressing those risks should be the focus.” (DrinkWise, industry-funded NGO)<br><br>“WHO also points to the average per capita consumption and establishes a direct relationship of alcohol consumption with damage to health, while reality shows that there are specific groups, whether by age or unhealthy habits of life (high in sugars and fats, spirits consumption and high intake episodes alcoholic) those whose (sic.) are scientifically related to major health problems” (Vinos de Chile, private sector entity)	Alcohol Awareness Foundation Ireland (trading as Drinkaware)<br>Alcohol Beverages Australia<br>Asociación Dominicana de Productores de Ron (ADOPRON)<br>AssoBirra<br>Association for Alcohol Responsibility and Education (aware.org)<br>Association of Alcohol Manufacturers and Importers<br>Australian Grape and Wine Inc. Australian Grape & Wine)<br>Beer Canada<br>Beer Institute<br>Belgian Brewers<br>Brazilian Beer Trade Association (SINDICERV)<br>Bundesverband der Deutschen Spirituosen-Industrie und -Importeure e.V. (BSI)/Federal Association of the German Spirits Industry and Importers (BSI)<br>Caribbean Breweries Association (CBA)<br>CEEV, Comité européen des entreprises vins<br>Cerveceros de España<br>Cerveceros Latinoamericanos<br>CTA – Confederation of Business Associations of Mozambique<br>Distilled Spirits Council of the United Sates<br>Drinks Ireland<br>DrinkWise<br>Educ'alcool<br>FEDERACIÓN ESPAÑOLA DEL VINO (FEV)<br>Fédération des Exportateurs de Vins et Spiritueux de France (FEVS)<br>FIVS<br>Fundación de Investigaciones Sociales A.C. (Foundation of Social Research)<br>International Alliance for Responsible Drinking (IARD)<br>ISWAI International Spirits & Wine Association of India<br>Japan Spirits & Liqueurs Makers Association (JSLMA)<br>Mexican Chamber of the Tequila Industry<br>México’s National Chamber of Beer and Malt<br>Regional Beverage Alcohol Alliance (RBAA)<br>Representantes-Importadores de Vinos y Licores Asociados (RIVLAS)<br>South African Liquor Brand owners Association<br>Spirits New Zealand, New Zealand Winegrowers and the Brewers Association of New Zealand<br>spiritsEUROPE<br>STIVA (Foundation for responsible alcohol consumption)<br>The Brewers of Europe

			The UK alcoholic drinks trade associations: British Beer & Pub Association, National Association of Cider Makers, Scotch Whisky Association and Wine and Spirit Trade Association<br>Trinidad & Tobago Beverage Alcohol Alliance (TTBAA)<br>Vinos de Chile<br>West Indies Rum & Spirits Producers Association (WIRSPA)<br>World Spirits Alliance<br>Worldwide Brewing Alliance
	Negative health, social and economic consequences for wider society	"Recognising and applauding the fact that WHO defines health broadly, we note that the adverse effects of alcohol are not limited to health, even in WHO's broad definition; alcohol consumption risks harm to the welfare not only of the drinker but also of others, and can impede sustainable social and economic development." (Kettil Bruun Society for Social and Epidemiological Research on Alcohol, NGO)<br><br>"In this context, alcohol's harm to others could become a crucial dimension to unlock synergies across different policy areas by facilitating the recognition of alcohol harm in the affected policy areas. This recognition of the real harm of alcohol in turn fosters understanding of the need for alcohol policy solutions to achieve sustainable outcomes in affected policy areas." (IOGT, NGO)	**Member States and governmental institutions**<br>Federal Office for Public Health, Switzerland<br>FPS Public health, Food chain safety and Environment, Belgium<br>Guyana Mission<br>Ministerio de Salud Pública de la República de Cuba<br>Ministry of agriculture, Latvia<br>Ministry of Health, New Zealand<br>Ministry of Health, Republic of Slovenia<br>Ministry of Health, Welfare and Sport, The Netherlands<br>Ministry of Public Health, Thailand<br>Ministry of Social Affairs of Estonia<br>**UN system and other IGOs**<br>European Centre Social Welfare Policy and Research<br>UNDP<br>United Nations Conference on Trade and Development<br>**Academic institutions**<br>MRC/CSO Social and Public Health Sciences Unit, University of Glasgow<br>School of Public Health, LKS Faculty of Medicine, The University of Hong Kong<br>SHORE Research Centre<br>**NGOs**<br>Abstinentenverband des Kantons Zürich<br>AESKAN<br>Alcohol & Drug Information Centre (ADIC), India<br>Alcohol Action Ireland<br>Alcohol Action New Zealand<br>Alcohol and Drug Information Centre (ADIC)<br>Alcohol Focus Scotland<br>Alcohol Health Alliance<br>Amardeep India<br>APABurkina<br>Association for Promoting Social Action (APSA)

			Balance, the North East Alcohol Office<br>Canadian Centre for Substance use and Addiction (CCSA)<br>EHYT Finnish Association for Substance Abuse Prevention<br>European Alcohol Policy Alliance<br>FORUT<br>Foundation for Alcohol Research and Education<br>Foundation for Innovative Social Development (FISD)<br>Global Alcohol Policy Alliance<br>Hāpai Te Hauora Tapui Limited<br>Healthy India Alliance<br>Hope and Beyond<br>HRIDAY<br>Humankind Charity<br>Institute for Research and Development "Utrip"<br>Institute of Alcohol Studies<br>International Blue Cross<br>International Federation of Medical Students' Association (IFMSA)<br>International Youth Health Organizations<br>IOGT Germany<br>IOGT Guinea-Bissau<br>IOGT Iceland<br>IOGT International<br>IOGT Norway<br>IOGT Switzerland<br>IOGT-NTO<br>Junis<br>Juvente<br>Juvente Switzerland<br>Kettil Bruun Society for Social and Epidemiological Research on Alcohol<br>Liberia Alcohol Policy Alliance<br>Lithuanian Tobacco and Alcohol Control Coalition<br>McCabe Centre for Law & Cancer<br>movendi slovakia<br>Nada India Foundation<br>National Alliance for Action on Alcohol<br>NCD Alliance<br>Newcastle Coalition inner city resident groups, small businesses and concerned citizens<br>Nigeria Alcohol Prevention Youth Initiative

			Nordic Alcohol and Drug Policy Network (NordAN)<br>Núll Prósent Hreyfingin<br>Recovery And Humanitarian Action Management Agency (RAHAMA)<br>Research and Training Center for Community Development (RTCCD)- The coordination organization of the Vienam Non-Communicable Diseases Control and Prevention Alliance (NCDs-VN)<br>Scottish Health Action on Alcohol Problems - SHAAP<br>Senegalese Alcohol Policy Alliance (SenAPA)<br>Sierra Leone Alcohol Policy Alliance (SLAPA)<br>Southern African Alcohol Policy Alliance<br>Sri Lanka Medical Association<br>Students Campaign Against Drugs<br>Tanzania Media Women's Association (TAMWA)<br>The Wellbeing Initiative<br>United States Alcohol Policy Alliance (U.S.APA)<br>Vision for Alternative Development<br>WAAPA-BENIN/ Secrétariat (Initiative pour l'Education et le Contrôle du Tabagisme)<br>West African Alcohol Policy Alliance (WAAPA)<br>World Federation Against Drugs<br>Youth against Alcoholism and Drug Dependency (YADD)
