# Supplementary material for: A Framing Analysis of Consultation Submissions on the WHO Global Strategy to Reduce the Harmful Use of Alcohol: Values and Interests
Source: Int J Health Policy Manag. 2021 Jun 26;11(8):1550–61. doi: 10.34172/ijhpm.2021.68 (PMC9808336; doi:10.34172/ijhpm.2021.68)
Supplement: Supplementary file 3 — Identified Framing Assigning Causation. [file ijhpm-11-1550-s003.pdf]

Article title: A Framing Analysis of Consultation Submissions on the WHO Global Strategy to Reduce the Harmful Use of Alcohol: Values and Interests

Journal name: International Journal of Health Policy and Management (IJHPM)

Authors' information: Chiara Rinaldi*[1], May CI van Schalkwyk[1], Matt Egan[2], Mark Petticrew[2]

[1]Department of Health Services Research and Policy, London School of Hygiene and Tropical Medicine, London, UK.

[2]Department of Public Health, Environments and Society, London School of Hygiene and Tropical Medicine, London, UK.

(*corresponding author: chiara.rinaldi@lshtm.ac.uk)

Supplementary file 3. Identified Framing Assigning Causation

Table S3: Expanded table of the identified frames, submitting stakeholders and exemplifying quotes.

Frames		Quotes	Stakeholders
**Individual choice**	Alcohol misuse is caused by uninformed individual choices	"For general consumers, the priority is to ensure that the consumers are best informed about the choices they make. This involves knowing more about the products they are consuming as well as the impact of various consumption patterns." (Drinks Ireland, trade association)<br><br>"The proportion of people now abstaining from alcohol consumption has increased by 34%. This is a dramatic increase indicating that the Australians are making their own choices when it comes to alcohol consumption." (Australian Wine and Grape Inc., trade association)	**Member States and governmental institutions**<br>Ministerio de salud y proteccion social de Colombia<br>Ministry of agriculture, Latvia<br>Ministry of Social Affairs of Estonia<br>Permanent Representation of Italy to the International Organizations<br>**Non-governmental organisations (NGOs)**<br>AFGHANISTAN GREEN CRESCENT ORGANIZAION (AGCO)<br>IOGT-VN<br>**Private sector entities**<br>Alcohol Awareness Foundation Ireland (trading as Drinkaware)<br>Alcohol Beverages Australia<br>AssoBirra<br>Australian Grade and Wine Inc.<br>Beer Canada<br>Beer Institute<br>Belgian Brewers<br>Brazilian Beer Trade Association (SINDICERV)<br>Bundesverband der Deutschen Spirituosen-Industrie und -Importeure e.V. (BSI)/<br>Federal Association of the German Spirits Industry and Importers (BSI)

			Caribbean Breweries Association (CBA)<br>CEEV, Comité européen des entreprises vins<br>CTA – Confederation of Business Associations of Mozambique<br>Drinks Ireland<br>DrinkWise<br>Educ'alcool<br>FEDERACIÓN ESPAÑOLA DEL VINO (FEV)<br>Fédération des Exportateurs de Vins et Spiritueux de France (FEVS)<br>Fundación de Investigaciones Sociales A.C. (Foundation of Social Research)<br>International Alliance for Responsible Drinking (IARD)<br>ISWAI International Spirits & Wine Association of India (Board Of Directors have approved name change to India Spirits & Wines Association Incorporated - application being made to Registrar Of Companies)<br>México's National Chamber of Beer and Malt<br>Regional Beverage Alcohol Alliance (RBAA)<br>Spirits New Zealand, New Zealand Winegrowers and the Brewers Association of New Zealand<br>spiritsEUROPE<br>STIVA (Foundation for responsible alcohol consumption)<br>The UK alcoholic drinks trade associations: British Beer & Pub Association, National Association of Cider Makers, Scotch Whisky Association and Wine and Spirit Trade Association<br>Trinidad & Tobago Beverage Alcohol Alliance (TTBAA)<br>Vinos de Chile<br>World Spirits Alliance<br>Worldwide Brewing Alliance
**Social norms**	Alcohol misuse is a deep-rooted social norm	"Another setback is the social role alcohol plays which makes it difficult to educate people on its harmful effect." (Association of Advocates against Alcohol Harm in Nigeria, NGO)<br><br>"The ultimate challenge, to cite the Discussion Paper, is that "Alcohol consumption is embedded in social norms and traditions", which makes it a more complex and difficult behaviour	**Member States and governmental institutions**<br>Ministerio de salud y proteccion social de Colombia<br>Ministry of Health of the Czech Republic<br>Permanent Mission of Georgia to the United Nations Office in Geneva and other international organizations<br>Spanish Ministry of Health, Consumer Affairs and Welfare<br>United States of America<br>**Academic institutions**<br>MRC/CSO Social and Public Health Sciences Unit, University of Glasgow<br>**NGOs**<br>APABurkina<br>Association of Advocates against Alcohol Harm in Nigeria<br>Canadian Centre for Substance use and Addiction (CCDA)

		to change.” (Drinkaware, industry-funded charity)<br><br>“Variations in social norms and acceptability affect perceptions of what constitutes excessive drinking, which may deter or hinder public health efforts to reduce harmful consumption of alcohol. Youth may in particular be affected by social norms tacitly suggesting approval of underage or excessive consumption.” (United States of America, Member State)	Centre for Alcohol Studies, Thai Health Promotion Foundation<br>FORUT<br>Green Crescent Zimbabwe<br>Green Moon<br>HealthBridge Foundation of Canada, Vietnam Office<br>Liberia Alcohol Policy Alliance<br>NCD Alliance<br>Recovery And Humanitarian Action Management Agency (RAHAMA)<br>RECOVERY, z.s.<br>Senegalese Alcohol Policy Alliance (SenAPA)<br>Sierra Leone Alcohol Policy Alliance (SLAPA)<br>Stopdrink Network<br>WAAPA-BENIN/ Secrétariat (Initiative pour l'Education et le Contrôle du Tabagisme)<br>West African Alcohol Policy Alliance (WAAPA)<br>**Private sector entities**<br>Alcohol Awareness Foundation Ireland (trading as Drinkaware)<br>AssoBirra<br>Association for Alcohol Responsibility and Education (aware.org)<br>Australian Grape and Wine Inc. (Australian Grape & Wine)<br>Beer Canada<br>Brazilian Beer Trade Association (SINDICERV)<br>Caribbean Breweries Association (CBA)<br>CEEV, Comité européen des entreprises vins<br>Cerveceros de España<br>DrinkWise<br>FEDERACIÓN ESPAÑOLA DEL VINO (FEV)<br>Fédération des Exportateurs de Vins et Spiritueux de France (FEVS)<br>ISWAI International Spirits & Wine Association of India<br>Regional Beverage Alcohol Alliance (RBAA)<br>STIVA (Foundation for responsible alcohol consumption)<br>The Brewers of Europe<br>Trinidad & Tobago Beverage Alcohol Alliance (TTBAA)<br>Vinos de Chile
**Under-regulation**	Alcohol misuse is caused by the widespread and underregulated	“Over the past decade, the world has not seen progress regarding alcohol prevention and control: neither in reducing alcohol consumption and	**Member States and governmental institutions**<br>Department of Health, Ireland<br>Directorate of Health, Iceland<br>Federal Office for Public Health, Switzerland<br>Guyana Mission

	availability, affordability and marketing of alcohol	related harm, nor in increasing alcohol policy best buy implementation.” (IOGT, NGO)<br><br>“The pervasive cultural views of alcohol consumption as a social and cultural norm in countries where alcohol is consumed giver (sic.) rise to the persistent belief that people should be “free to choose” without government shaping when, where and how much alcohol they consume.” (NCD Alliance, NGO)<br><br>“The considerable resources and consolidation of global alcohol companies enables them to interfere with many attempts of member states to achieve the implementation of effective legislation to reduce alcohol-related harm (…). A recent example of intensive industry involvement in policy making took place in Vietnam where an initial draft of legislation included the best buys and the final version was considerably weaker.” (SHORE Research Centre, academic institution)	Instituto sobre Alcoholismo y Fármacodependencia (IAFA), Costa Rica<br>Ministerio de Salud Pública de la República de Cuba<br>Ministerio de salud y proteccion social de Colombia<br>Ministry of Health of the Czech Republic<br>Ministry of Health, National Commission Against Addictions, Mexico<br>Ministry of Health, New Zealand<br>Ministry of Health, Republic of Slovenia<br>Ministry of Health, Welfare and Sport, The Netherlands<br>Ministry of Public Health, Thailand<br>Ministry of Social Affairs of Estonia<br>NCPHA, MoH Bulgaria<br>Permanent Mission of Georgia to the United Nations Office in Geneva and other international organizations<br>South African Medical Research Council<br>Spanish Ministry of Health, Consumer Affairs and Welfare<br>The National Institute of Public Health, Czech Republic<br>The State Agency for Prevention of Alcohol Related Problems, Poland<br>**UN system and other international organisations (IGOs)**<br>The Pacific Community (SPC) (on behalf of Pacific Island Countries and Territories)<br>UNDP<br>**Academic institutions**<br>Community Action on Youth and Drugs National Coordination Team, Massey University<br>MRC/CSO Social and Public Health Sciences Unit, University of Glasgow<br>School of Public Health, LKS Faculty of Medicine, The University of Hong Kong<br>SHORE Research Centre<br>SPECTRUM (Shaping Public hEalth poliCies To Reduce IneqUalities and harM)<br>TRAPS (Transformative Research on Alcohol Policy and Science programme at the University of York)<br>**NGOs**<br>Abstinentenverband des Kantons Zürich<br>AESKAN<br>Alcohol & Drug Information Centre (ADIC), India<br>Alcohol Action Ireland<br>Alcohol Action New Zealand<br>Alcohol and Drug Information Centre (ADIC)<br>Alcohol Focus Scotland<br>Alcohol Health Alliance<br>Amardeep India

			APABurkina<br>Asia Pacific Alcohol Policy Alliance<br>Association for Promoting Social Action (APSA)<br>Association of Advocates against Alcohol Harm in Nigeria<br>Australasian Professional Society on Alcohol and other Drugs (APSAD)<br>Balance, the North East Alcohol Office<br>Canadian Centre for Substance use and Addiction (CCSA)<br>Cancer Society<br>Centre for Alcohol Studies, Thai Health Promotion Foundation<br>CROISSANT VERT NIGERIEN(CVN)<br>Cruz Azul no Brasil<br>EHYT Finnish Association for Substance Abuse Prevention<br>European Alcohol Policy Alliance<br>European Mutual help Network for Alcohol related problems (EMNA)<br>Fondacioni YESILAY<br>FORUT<br>Foundation for Alcohol Research and Education<br>Global Alcohol Policy Alliance<br>Green crescent of Congo est<br>Green Crescent Society, Turkey<br>Green Crescent South Africa<br>Green Crescent Zimbabwe<br>Green Moon<br>Hāpai Te Hauora Tapui Limited<br>HealthBridge Foundation of Canada, Vietnam Office<br>Healthy India Alliance<br>Hong Kong Alliance for Advocacy Against Alcohol<br>Hope and Beyond<br>HRIDAY<br>Humankind Charity<br>Institute for Research and Development "Utrip"<br>Institute of Alcohol Studies<br>International Blue Cross<br>International Federation of Medical Students' Association (IFMSA)<br>International Youth Health Organizations<br>IOGT Germany<br>IOGT Guinea-Bissau<br>IOGT Iceland<br>IOGT International

			IOGT Norway<br>IOGT Switzerland<br>IOGT-NTO<br>Junis<br>Juvente<br>Juvente Switzerland<br>Kettil Bruun Society for Social and Epidemiological Research on Alcohol<br>Liberia Alcohol Policy Alliance<br>Lithuanian Tobacco and Alcohol Control Coalition<br>Moroccan Green Crescent<br>movendi slovakia<br>Nada India Foundation<br>National Alliance for Action on Alcohol<br>NCD Alliance<br>Newcastle Coalition inner city resident groups, small businesses and concerned citizens<br>Nigeria Alcohol Prevention Youth Initiative<br>Nordic Alcohol and Drug Policy Network (NordAN)<br>Núll Prósent Hreyfingin<br>People Center for Development and Peace<br>Pioneer Total Abstinence Association<br>Recovery And Humanitarian Action Management Agency (RAHAMA)<br>Research and Training Center for Community Development (RTCCD)- The coordination organization of the Vienam Non-Communicable Diseases Control and Prevention Alliance (NCDs-VN)<br>Scottish Health Action on Alcohol Problems - SHAAP<br>Senegalese Alcohol Policy Alliance (SenAPA)<br>Serenity Harm Reduction Programme Zambia (SHARPZ)<br>Sierra Leone Alcohol Policy<br>Southern African Alcohol Policy Alliance Alliance (SLAPA)<br>Sri Lanka Medical Association<br>Stopdrink Network<br>Students Campaign Against Drugs<br>Swedish cancer society<br>Tanzania Network Against Alcohol Abuse - TAAnet<br>The Wellbeing Initiative<br>Trimbos Institute<br>UDRUZENJE GRADANA ZELENI POLUMJESEC U BIH<br>Uganda Youth Development Link

			United States Alcohol Policy Alliance (U.S.APA)<br>Vision for Alternative Development<br>WAAPA-BENIN/ Secrétariat (Initiative pour l'Education et le Contrôle du Tabagisme)<br>West African Alcohol Policy Alliance (WAAPA)<br>World Federation Against Drugs<br>Youth against Alcoholism and Drug Dependency (YADD)
