# Supplementary material for: A Framing Analysis of Consultation Submissions on the WHO Global Strategy to Reduce the Harmful Use of Alcohol: Values and Interests
Source: Int J Health Policy Manag. 2021 Jun 26;11(8):1550–61. doi: 10.34172/ijhpm.2021.68 (PMC9808336; doi:10.34172/ijhpm.2021.68)
Supplement: Supplementary file 4 — Identified Framing Proposing Solutions. [file ijhpm-11-1550-s004.pdf]

Article title: A Framing Analysis of Consultation Submissions on the WHO Global Strategy to Reduce the Harmful Use of Alcohol: Values and Interests

Journal name: International Journal of Health Policy and Management (IJHPM)

Authors' information: Chiara Rinaldi*[1], May CI van Schalkwyk[1], Matt Egan[2], Mark Petticrew[2]

[1]Department of Health Services Research and Policy, London School of Hygiene and Tropical Medicine, London, UK.

[2]Department of Public Health, Environments and Society, London School of Hygiene and Tropical Medicine, London, UK.

(*corresponding author: chiara.rinaldi@lshtm.ac.uk)

Supplementary file 4. Identified Framing Proposing Solutions

Table S4: Expanded table of the identified frames, submitting stakeholders and exemplifying quotes.

Frames		Quotes	Stakeholders
**Targeted interventions versus population-level regulation**	Broad "menu" of policy options targeted at at-risk individuals or risky behaviours	"FIVS has not seen much success with "whole population measures" which entail restrictive policies affecting all who consume alcohol, the vast majority of whom do so responsibly. FIVS and its members have focused efforts instead on the small, and declining minority, whose intake regularly exceeds recommended levels." (FIVS, trade association)<br><br>"Responsible alcohol consumption is based on informed consumers and professional producers. Therefore, developing of the next period of the Action Plan for Reducing Alcohol Consumption and Reducing Alcoholism, more emphasis should be placed on public awareness campaigns and campaigns, that talk about responsible drinking, thus reducing alcohol overuse or risky use in society." (Ministry of Agriculture Latvia, Member State)<br><br>"Heaviest drinkers, including heavy episodic drinkers, are the least sensitive to	**Member States and governmental institutions**<br>Guyana Mission<br>Ministerio de salud y proteccion social de Colombia<br>Ministry of agriculture, Latvia<br>Permanent Representation of Italy to the International Organizations<br>United States of America<br>**UN system and other international organisations (IGOs)**<br>United Nations Conference on Trade and Development<br>**Non-governmental organisations (NGOs)**<br>Green Crescent South Africa<br>Green Crescent Zimbabwe<br>Green Crescents Kazakhstan<br>Italian Society on Alcohol (SIA)<br>Public Union against Bad Habits<br>value health africa<br>**Private sector entities**<br>Alcohol Awareness Foundation Ireland (trading as Drinkaware)<br>Alcohol Beverages Australia<br>Asociación Dominicana de Productores de Ron (ADOPRON)<br>AssoBirra<br>Association for Alcohol Responsibility and Education (aware.org)<br>Australian Grape and Wine Inc. Australian Grape & Wine)<br>Beer Canada<br>Beer Institute<br>Belgian Brewers<br>Brazilian Beer Trade Association (SINDICERV)

		pricing policies. To be effective, a regulatory framework, which includes taxation, must be accompanied by interventions aimed specifically at harmful drinking (WHO, 2010).” (Brazilian Beer Trade Association, trade association)	Bundesverband der Deutschen Spirituosen-Industrie und -Importeure e.V. (BSI)/Federal Association of the German Spirits Industry and Importers (BSI)<br>Caribbean Breweries Association (CBA)<br>CEEV, Comité européen des entreprises vins<br>Cerveceros de España<br>Cerveceros Latinoamericanos<br>CTA – Confederation of Business Associations of Mozambique<br>Distilled Spirits Council of the United Sates<br>Drinks Ireland<br>DrinkWise<br>Educ'alcool<br>FEDERACIÓN ESPAÑOLA DEL VINO (FEV)<br>Fédération des Exportateurs de Vins et Spiritueux de France (FEVS)<br>FIVS<br>Fundación de Investigaciones Sociales A.C. (Foundation of Social Research)<br>International Alliance for Responsible Drinking (IARD)<br>ISWAI International Spirits & Wine Association of India<br>Japan Spirits & Liqueurs Makers Association (JSLMA)<br>Mexican Chamber of the Tequila Industry<br>México’s National Chamber of Beer and Malt<br>Regional Beverage Alcohol Alliance (RBAA)<br>Representantes-Importadores de Vinos y Licores Asociados (RIVLAS)<br>South African Liquor Brand owners Association<br>Spirits New Zealand, New Zealand Winegrowers and the Brewers Association of New Zealand<br>spiritsEUROPE<br>STIVA (Foundation for responsible alcohol consumption)<br>The Brewers of Europe<br>The UK alcoholic drinks trade associations: British Beer & Pub Association, National Association of Cider Makers, Scotch Whisky Association and Wine and Spirit Trade Association<br>Trinidad & Tobago Beverage Alcohol Alliance (TTBAA)<br>Vinos de Chile<br>West Indies Rum & Spirits Producers Association (WIRSPA)<br>World Spirits Alliance<br>Worldwide Brewing Alliance
	Universal implementation of the three ‘best buys’ at population level	“Over the last decade, WHO and health researchers confirmed the relevance of the so called ‘best buys’ to cost-effectively reduce and prevent alcohol related harm. Therefore, continuation of these policy directions should be envisaged.” (European Alcohol Policy Alliance, NGO)<br><br>“The most effective ways to reduce alcohol harms, such as increasing price or reducing availability require market intervention, and the lack of progress in	**Member States and governmental institutions**<br>Cook Islands Ministry of Health<br>Department of Health, Ireland<br>Directorate of Health, Iceland<br>Federal Office for Public Health, Switzerland<br>Instituto sobre Alcoholismo y Fármacodependencia (IAFA), Costa Rica<br>Ministerio de Salud Pública de la República de Cuba<br>Ministerio de salud y proteccion social de Colombia<br>Ministry of Health Mozambique<br>Ministry of Health of the Czech Republic<br>Ministry of Health of the Republic of Latvia<br>Ministry of Health, National Commission Against Addictions, Mexico<br>Ministry of Health, New Zealand

		implementation of measures such as these may largely depend on progress in promoting the principle that market regulation is fundamental to effective alcohol policy.” (TRAPS, University of York, academic institution)	Ministry of Health, Republic of Slovenia<br>Ministry of Health, Welfare and Sport, The Netherlands<br>Ministry of Public Health, Thailand<br>Ministry of Social Affairs of Estonia<br>NCPHA, MoH Bulgaria<br>Permanent Mission of Georgia to the United Nations Office in Geneva and other international organizations<br>South African Medical Research Council<br>The National Institute of Public Health, Czech Republic<br>The State Agency for Prevention of Alcohol Related Problems, Poland<br>**UN system and other IGOs**<br>The Pacific Community (SPC) (on behalf of Pacific Island Countries and Territories)<br>UNDP<br>**Academic institutions**<br>Community Action on Youth and Drugs National Coordination Team, Massey University<br>MRC/CSO Social and Public Health Sciences Unit, University of Glasgow<br>School of Public Health, LKS Faculty of Medicine, The University of Hong Kong<br>SHORE Research Centre<br>SPECTRUM (Shaping Public hEalth poliCies To Reduce IneqUalities and harM)<br>TRAPS (Transformative Research on Alcohol Policy and Science programme at the University of York)<br>**NGOs**<br>Abstinentenverband des Kantons Zürich<br>AESKAN<br>Alcohol & Drug Information Centre (ADIC), India<br>Alcohol Action Ireland<br>Alcohol Action New Zealand<br>Alcohol and Drug Information Centre (ADIC)<br>Alcohol Focus Scotland<br>Alcohol Health Alliance<br>Amardeep India<br>APABurkina<br>Asia Pacific Alcohol Policy Alliance<br>Association for Promoting Social Action (APSA)<br>Australasian Professional Society on Alcohol and other Drugs (APSAD)<br>Canadian Centre for Substance use and Addiction (CCSA)<br>Cancer Society<br>Centre for Alcohol Studies, Thai Health Promotion Foundation<br>Cruz Azul no Brasil<br>EHYT Finnish Association for Substance Abuse Prevention<br>European Alcohol Policy Alliance<br>European Mutual help Network for Alcohol related problems (EMNA)<br>Fondacioni YESILAY<br>FORUT<br>Foundation for Alcohol Research and Education<br>Foundation for Innovative Social Development (FISD)<br>Global Alcohol Policy Alliance<br>Hāpai Te Hauora Tapui Limited<br>Healthy India Alliance

			Hong Kong Alliance for Advocacy Against Alcohol<br>Hope and Beyond<br>HRIDAY<br>Humankind Charity<br>Institute for Research and Development "Utrip"<br>Institute of Alcohol Studies<br>International Federation of Medical Students' Association (IFMSA)<br>International Youth Health Organizations<br>IOGT Germany<br>IOGT Guinea-Bissau<br>IOGT Iceland<br>IOGT International<br>IOGT Norway<br>IOGT Switzerland<br>IOGT-NTO<br>Junis<br>Juvente<br>Juvente Switzerland<br>Liberia Alcohol Policy Alliance<br>Lithuanian Tobacco and Alcohol Control Coalition<br>McCabe Centre for Law & Cancer<br>Moroccan Green Crescent<br>movendi slovakia<br>Nada India Foundation<br>National Alliance for Action on Alcohol<br>NCD Alliance<br>Newcastle Coalition inner city resident groups, small businesses and concerned citizens<br>Nigeria Alcohol Prevention Youth Initiative<br>Nordic Alcohol and Drug Policy Network (NordAN)<br>Núll Prósent Hreyfingin<br>People Center for Development and Peace<br>RECOVERY, z.s.<br>Research and Training Center for Community Development (RTCCD)- The coordination organization of the Vienam Non-Communicable Diseases Control and Prevention Alliance (NCDs-VN)<br>Scottish Health Action on Alcohol Problems - SHAAP<br>Senegalese Alcohol Policy Alliance (SenAPA)<br>Sierra Leone Alcohol Policy Alliance (SLAPA)<br>Southern African Alcohol Policy Alliance<br>Students Campaign Against Drugs<br>Swedish cancer society<br>The Wellbeing Initiative<br>UDRUZENJE GRADANA ZELENI POLUMJESEC U BIH<br>Uganda Youth Development Link<br>United States Alcohol Policy Alliance (U.S.APA)<br>Vision for Alternative Development<br>WAAPA-BENIN/ Secrétariat (Initiative pour l'Education et le Contrôle du Tabagisme)<br>West African Alcohol Policy Alliance (WAAPA)

			World Federation Against Drugs<br>Youth against Alcoholism and Drug Dependency (YADD)
**Local versus global solutions**	Local action specific to the national context (no "one size fits all")	"Where improvements have been made, a crucial element has been the recognition that harmful use of alcohol often requires tailored, local and culturally-sensitive measures." (AssoBirra, trade association)<br><br>"The situation is not the same in all countries and in all societies. Cultural, social and economic characteristics are not the same everywhere and consumption patterns, such as genetic components, absolutely do not allow the generalization of situations from one country to another, from one people to another, from one culture to another and from one consumption model to another." (Educ'alcool, industry-funded NGO)<br><br>"Our experience over the past two years has highlighted the fact that South Africa given its own unique set of challenges requires not only targeted programmes but education and awareness interventions in a 'nuanced and contextual' manner to address the harmful consumption of alcohol – its situational variables and challenges differ to other countries. (…) A one-size fits all approach cannot be implemented without due consideration for the regional and national settings." (Association for Alcohol Responsibility and Education, social aspect organisation)	**Member States and governmental institutions**<br>Ministry of agriculture, Latvia<br>Permanent Representation of Italy to the International Organizations<br>United States of America<br>**NGOs**<br>CROISSANT VERT NIGERIEN(CVN)<br>People Center for Development and Peace<br>**Private sector entities**<br>Alcohol Beverages Australia<br>Asociación Dominicana de Productores de Ron (ADOPRON)<br>AssoBirra<br>Association for Alcohol Responsibility and Education (aware.org)<br>Australian Grape and Wine Inc. Australian Grape & Wine)<br>Beer Canada<br>Beer Institute<br>Brazilian Beer Trade Association (SINDICERV)<br>Caribbean Breweries Association (CBA)<br>CEEV, Comité européen des entreprises vins<br>Cerveceros de España<br>CTA – Confederation of Business Associations of Mozambique<br>Distilled Spirits Council of the United Sates<br>Educ'alcool<br>FEDERACIÓN ESPAÑOLA DEL VINO (FEV)<br>Fédération des Exportateurs de Vins et Spiritueux de France (FEVS)<br>FIVS<br>Fundación de Investigaciones Sociales A.C. (Foundation of Social Research)<br>International Alliance for Responsible Drinking (IARD)<br>Japan Spirits & Liqueurs Makers Association (JSLMA)<br>Mexican Chamber of the Tequila Industry<br>México's National Chamber of Beer and Malt<br>Regional Beverage Alcohol Alliance (RBAA)<br>Representantes-Importadores de Vinos y Licores Asociados (RIVLAS)<br>South African Liquor Brand owners Association<br>Spirits New Zealand, New Zealand Winegrowers and the Brewers Association of New Zealand<br>spiritsEUROPE<br>The Brewers of Europe<br>The UK alcoholic drinks trade associations: British Beer & Pub Association, National Association of Cider Makers, Scotch Whisky Association and Wine and Spirit Trade Association<br>Trinidad & Tobago Beverage Alcohol Alliance (TTBAA)<br>West Indies Rum & Spirits Producers Association (WIRSPA)<br>World Spirits Alliance

			Worldwide Brewing Alliance
	Global action to address cross-border issues and industry interference	“We also see a great value in international cooperation in any form to address the cross-border issues of alcohol policy. Globalization and digitalization create new challenges and can easily dilute individual countries’ efforts to tackle alcohol-related harm. The issue is particularly relevant in the area of marketing where the global nature of media puts the effectiveness of national regulations into question.” (Ministry of Social Affairs of Estonia, Member State).<br><br>“The implementation of Global Strategy has been patchy and, in some instances, suffering from regulatory freeze, due to legally binding trade agreements. Given the harm caused by alcohol related harm, alcohol policy should get the attention and legally binding framework it deserves, to ensure effective implementation of SDG target 3.4 and 3.5.” (European Alcohol Policy Alliance, NGO)	**Member States and governmental institutions**<br>Centre for Diseases Prevention and Control, Latvia<br>Cook Islands Ministry of Health<br>Department of Health, Ireland<br>Directorate of Health, Iceland<br>Federal Office for Public Health, Switzerland<br>FPS Public health, Food chain safety and Environment, Belgium<br>Guyana Mission<br>Ministry of Health of the Czech Republic<br>Ministry of Health, National Commission Against Addictions, Mexico<br>Ministry of Health, New Zealand<br>Ministry of Health, Republic of Slovenia<br>Ministry of Health, Welfare and Sport, The Netherlands<br>Ministry of Public Health, Thailand<br>Ministry of Social Affairs of Estonia<br>NCPHA, MoH Bulgaria<br>South African Medical Research Council<br>Spanish Ministry of Health, Consumer Affairs and Welfare<br>**UN system and other IGOs**<br>The Pacific Community (SPC) (on behalf of Pacific Island Countries and Territories)<br>UNDP<br>**Academic institutions**<br>Community Action on Youth and Drugs National Coordination Team, Massey University<br>MRC/CSO Social and Public Health Sciences Unit, University of Glasgow<br>SHORE Research Centre<br>SPECTRUM (Shaping Public hEalth poliCies To Reduce IneqUalities and harM)<br>TRAPS (Transformative Research on Alcohol Policy and Science programme at the University of York)<br>**NGOs**<br>Abstinentenverband des Kantons Zürich<br>AESKAN<br>Alcohol & Drug Information Centre (ADIC), India<br>Alcohol Action Ireland<br>Alcohol Action New Zealand<br>Alcohol and Drug Information Centre (ADIC)<br>Alcohol Focus Scotland<br>Alcohol Health Alliance<br>Alcohol Policy Alliance Gambia<br>Amardeep India<br>APABurkina<br>Asia Pacific Alcohol Policy Alliance<br>Australasian Professional Society on Alcohol and other Drugs (APSAD)<br>Balance, the North East Alcohol Office<br>Canadian Centre for Substance use and Addiction (CCSA)<br>Cancer Society

			Centre for Alcohol Studies, Thai Health Promotion Foundation<br>EHYT Finnish Association for Substance Abuse Prevention<br>European Alcohol Policy Alliance<br>European Mutual help Network for Alcohol related problems (EMNA)<br>FORUT<br>Foundation for Alcohol Research and Education<br>Foundation for Innovative Social Development (FISD)<br>Global Alcohol Policy Alliance<br>Green Crescent Society, Turkey<br>Green Crescents Kazakhstan<br>Hāpai Te Hauora Tapui Limited<br>HealthBridge Foundation of Canada, Vietnam Office<br>Healthy India Alliance<br>Hong Kong Alliance for Advocacy Against Alcohol<br>Hope and Beyond<br>HRIDAY<br>Institute for Research and Development "Utrip"<br>Institute of Alcohol Studies<br>International Blue Cross<br>International Federation of Medical Students' Association (IFMSA)<br>International Youth Health Organizations<br>IOGT Gambia<br>IOGT Germany<br>IOGT Guinea-Bissau<br>IOGT Iceland<br>IOGT International<br>IOGT Norway<br>IOGT Switzerland<br>IOGT-NTO<br>Junis<br>Juvente<br>Juvente Switzerland<br>Kettil Bruun Society for Social and Epidemiological Research on Alcohol<br>Liberia Alcohol Policy Alliance<br>Lithuanian Tobacco and Alcohol Control Coalition<br>McCabe Centre for Law & Cancer<br>movendi slovakia<br>Nada India Foundation<br>National Alliance for Action on Alcohol<br>NCD Alliance<br>Newcastle Coalition inner city resident groups, small businesses and concerned citizens<br>Nigeria Alcohol Prevention Youth Initiative<br>Nordic Alcohol and Drug Policy Network (NordAN)<br>Núll Prósent Hreyfingin<br>Recovery And Humanitarian Action Management Agency (RAHAMA)<br>Research and Training Center for Community Development (RTCCD)- The coordination organization of the Vienam Non-Communicable Diseases Control and Prevention Alliance (NCDs-VN)

			Scottish Health Action on Alcohol Problems - SHAAP<br>Senegalese Alcohol Policy Alliance (SenAPA)<br>Sierra Leone Alcohol Policy Alliance (SLAPA)<br>Southern African Alcohol Policy Alliance<br>Sri Lanka Medical Association<br>Stopdrink Network<br>The Wellbeing Initiative<br>Trimbos Institute<br>UDRUZENJE GRADANA ZELENI POLUMJESEC U BIH<br>United States Alcohol Policy Alliance (U.S.APA)<br>Vision for Alternative Development<br>WAAPA-BENIN/ Secrétariat (Initiative pour l'Education et le Contrôle du Tabagisme)<br>West African Alcohol Policy Alliance (WAAPA)<br>World Federation Against Drugs<br>Youth against Alcoholism and Drug Dependency (YADD)<br>**Private sector entities**<br>Association of Alcohol Manufacturers and Importers<br>Belgian Brewers
**Partnership versus freedom from industry interference**	Involvement from a broad array of stakeholders, including industry	"This issue is not one that can be tackled by governments alone. Governments and international organisations need to work with industry and communities to develop evidence-based, targeted and effective initiatives to reduce harmful drinking" (Australian Grape and Wine Inc., trade association)<br><br>"The WHO and the GAS need to avoid an exclusionary viewpoint. The United Nations 2018 Political Declaration on Non-communicable Diseases, which calls for Member States to engage with the private sector for "its meaningful and effective contribution to the implementation of national responses to non-communicable diseases", should be the starting point for the WHO and the GAS." (Beer Canada, trade association)	**Member States and governmental institutions**<br>Guyana Mission<br>Ministerio de salud y proteccion social de Colombia<br>Permanent Representation of Italy to the International Organizations<br>United States of America<br>**UN system and other IGOs**<br>United Nations Conference on Trade and Development<br>**NGOs**<br>International Federation of Medical Students' Association (IFMSA)<br>**Private sector entities**<br>Alcohol Awareness Foundation Ireland (trading as Drinkaware)<br>Alcohol Beverages Australia<br>Asociación Dominicana de Productores de Ron (ADOPRON)<br>AssoBirra<br>Association for Alcohol Responsibility and Education (aware.org)<br>Association of Alcohol Manufacturers and Importers<br>Australian Grape and Wine Inc. Australian Grape & Wine)<br>Beer Canada<br>Beer Institute<br>Belgian Brewers<br>Brazilian Beer Trade Association (SINDICERV)<br>Bundesverband der Deutschen Spirituosen-Industrie und -Importeure e.V. (BSI)/Federal Association of the German Spirits Industry and Importers (BSI)<br>Caribbean Breweries Association (CBA)<br>CEEV, Comité européen des entreprises vins<br>Cerveceros de España<br>Cerveceros Latinoamericanos<br>CTA – Confederation of Business Associations of Mozambique<br>Distilled Spirits Council of the United Sates

			Drinks Ireland<br>DrinkWise<br>Educ'alcool<br>FEDERACIÓN ESPAÑOLA DEL VINO (FEV)<br>Fédération des Exportateurs de Vins et Spiritueux de France (FEVS)<br>FIVS<br>Fundación de Investigaciones Sociales A.C. (Foundation of Social Research)<br>International Alliance for Responsible Drinking (IARD)<br>ISWAI International Spirits & Wine Association of India<br>Japan Spirits & Liqueurs Makers Association (JSLMA)<br>Mexican Chamber of the Tequila Industry<br>México's National Chamber of Beer and Malt<br>Regional Beverage Alcohol Alliance (RBAA)<br>Representantes-Importadores de Vinos y Licores Asociados (RIVLAS)<br>South African Liquor Brand owners Association<br>Spirits New Zealand, New Zealand Winegrowers and the Brewers Association of New Zealand<br>spiritsEUROPE<br>STIVA (Foundation for responsible alcohol consumption)<br>The Brewers of Europe<br>The UK alcoholic drinks trade associations: British Beer & Pub Association, National Association of Cider Makers, Scotch Whisky Association and Wine and Spirit Trade Association<br>Trinidad & Tobago Beverage Alcohol Alliance (TTBAA)<br>Vinos de Chile<br>West Indies Rum & Spirits Producers Association (WIRSPA)<br>World Spirits Alliance<br>Worldwide Brewing Alliance
	The public sector should be independent from industry interference	"We are concerned about the undue influence of alcohol industry on the public health policy. The involvement of alcohol industry in the development of public health policy measures entails an inherent conflict of interest, which should be avoided." (Ministry of Social Affairs of Estonia, Member State)<br><br>"Countries need stronger support with technical capacity building regarding alcohol policy formulation, implementation, monitoring and safeguarding alcohol prevention and control efforts from the alcohol industry." (IOGT, NGO)<br><br>"The principles of the Global Strategy recognize that all involved parties have the	**Member States and governmental institutions**<br>Cook Islands Ministry of Health<br>Department of Health, Ireland<br>Federal Office for Public Health, Switzerland<br>Instituto sobre Alcoholismo y Fármacodependencia (IAFA), Costa Rica<br>Ministry of Health of the Czech Republic<br>Ministry of Health, National Commission Against Addictions, Mexico<br>Ministry of Health, New Zealand<br>Ministry of Health, Republic of Slovenia<br>Ministry of Health, Welfare and Sport, The Netherlands<br>Ministry of Social Affairs of Estonia<br>South African Medical Research Council<br>The National Institute of Public Health, Czech Republic<br>**UN system and other IGOs**<br>European Centre Social Welfare Policy and Research<br>The Pacific Community (SPC) (on behalf of Pacific Island Countries and Territories)<br>UNDP<br>**Academic institutions**<br>Community Action on Youth and Drugs National Coordination Team, Massey University<br>MRC/CSO Social and Public Health Sciences Unit, University of Glasgow<br>School of Public Health, LKS Faculty of Medicine, The University of Hong Kong

		responsibility not to undermine implementation of policies to reduce harmful use of alcohol, and that public health should be given deference in relation to competing interests. In practice, and given the alcohol industry's extensive engagement in efforts to undermine effective health policies across multiple jurisdictions, these principles risk being compromised by roles the Global Strategy envisages for economic operators." (SPECTRUM, academic institution)	SHORE Research Centre<br>SPECTRUM (Shaping Public hEalth poliCies To Reduce IneqUalities and harM)<br>TRAPS (Transformative Research on Alcohol Policy and Science programme at the University of York)<br>**NGOs**<br>Abstinentenverband des Kantons Zürich<br>AESKAN<br>Alcohol & Drug Information Centre (ADIC), India<br>Alcohol Action Ireland<br>Alcohol Action New Zealand<br>Alcohol and Drug Information Centre (ADIC)<br>Alcohol Focus Scotland<br>Amardeep India<br>APABurkina<br>Asia Pacific Alcohol Policy Alliance<br>Australasian Professional Society on Alcohol and other Drugs (APSAD)<br>Balance, the North East Alcohol Office<br>Canadian Centre for Substance use and Addiction (CCSA)<br>Cancer Society<br>Centre for Alcohol Studies, Thai Health Promotion Foundation<br>Cruz Azul no Brasil<br>EHYT Finnish Association for Substance Abuse Prevention<br>European Alcohol Policy Alliance<br>FORUT<br>Foundation for Alcohol Research and Education<br>Foundation for Innovative Social Development (FISD)<br>Global Alcohol Policy Alliance<br>Green Crescent Society, Turkey<br>Green Crescent South Africa<br>Green Crescent Zimbabwe<br>Hāpai Te Hauora Tapui Limited<br>HealthBridge Foundation of Canada, Vietnam Office<br>Healthy India Alliance<br>Hong Kong Alliance for Advocacy Against Alcohol<br>Hope and Beyond<br>HRIDAY<br>Humankind Charity<br>Institute for Research and Development "Utrip"<br>Institute of Alcohol Studies<br>International Blue Cross<br>International Youth Health Organizations<br>IOGT Gambia<br>IOGT Germany<br>IOGT Guinea-Bissau<br>IOGT Iceland<br>IOGT International<br>IOGT Norway<br>IOGT Switzerland

			IOGT-NTO<br>Junis<br>Juvente<br>Juvente Switzerland<br>Liberia Alcohol Policy Alliance<br>Lithuanian Tobacco and Alcohol Control Coalition<br>McCabe Centre for Law & Cancer<br>movendi slovakia<br>Nada India Foundation<br>National Alliance for Action on Alcohol<br>NCD Alliance<br>Newcastle Coalition inner city resident groups, small businesses and concerned citizens<br>Nigeria Alcohol Prevention Youth Initiative<br>Nordic Alcohol and Drug Policy Network (NordAN)<br>Núll Prósent Hreyfingin<br>People Center for Development and Peace<br>Research and Training Center for Community Development (RTCCD)- The coordination organization of the Vienam Non-Communicable Diseases Control and Prevention Alliance (NCDs-VN)<br>Scottish Health Action on Alcohol Problems - SHAAP<br>Senegalese Alcohol Policy Alliance (SenAPA)<br>Serenity Harm Reduction Programme Zambia (SHARPZ)<br>Sierra Leone Alcohol Policy Alliance (SLAPA)<br>Southern African Alcohol Policy Alliance<br>Sri Lanka Medical Association<br>Stopdrink Network<br>Students Campaign Against Drugs<br>Swedish cancer society<br>The Wellbeing Initiative<br>Trimbos Institute<br>Uganda Youth Development Link<br>United States Alcohol Policy Alliance (U.S.APA)<br>Vision for Alternative Development<br>WAAPA-BENIN/ Secrétariat (Initiative pour l'Education et le Contrôle du Tabagisme)<br>West African Alcohol Policy Alliance (WAAPA)<br>World Federation Against Drugs<br>Youth against Alcoholism and Drug Dependency (YADD)
