# Supplementary material for: A Framing Analysis of Consultation Submissions on the WHO Global Strategy to Reduce the Harmful Use of Alcohol: Values and Interests
Source: Int J Health Policy Manag. 2021 Jun 26;11(8):1550–61. doi: 10.34172/ijhpm.2021.68 (PMC9808336; doi:10.34172/ijhpm.2021.68)
Supplement: Supplementary file 5 — Identified Framing Justifying and Persuading. [file ijhpm-11-1550-s005.pdf]

Article title: A Framing Analysis of Consultation Submissions on the WHO Global Strategy to Reduce the Harmful Use of Alcohol: Values and Interests

Journal name: International Journal of Health Policy and Management (IJHPM)

Authors' information: Chiara Rinaldi*[1], May CI van Schalkwyk[1], Matt Egan[2], Mark Petticrew[2]

[1]Department of Health Services Research and Policy, London School of Hygiene and Tropical Medicine, London, UK.

[2]Department of Public Health, Environments and Society, London School of Hygiene and Tropical Medicine, London, UK.

(*corresponding author: chiara.rinaldi@lshtm.ac.uk)

Supplementary file 5. Identified Framing Justifying and Persuading

Table S5: Expanded table of the identified frames, submitting stakeholders and exemplifying quotes.

Frames		Quotes	Stakeholders
**Use of evidence**	To support own problem definitions, causes and solutions	"Duty increases have been evidenced to save lives and reduce harm: the most recent analysis from the Sheffield Alcohol Policy Model shows that raising alcohol duty above inflation for five successive years would reduce alcohol-related deaths by 5% and hospitalisations by 4%, averting over 600 fatalities a year." (Humankind Charity, NGO)<br><br>"One fact is indisputable: moderate alcohol users have a longer life expectancy than abstainers for life. With nuances and exceptions, it goes without saying, but this is amply demonstrated in general" (Educ'alcool, industry-funded charity, translated) **[This claim is not supported by the current scientific evidence base; no references were provided]**	**Member States and governmental institutions**<br>Cook Islands Ministry of Health<br>Ministry of Health, New Zealand<br>Ministry of Public Health, Thailand<br>Ministry of Social Affairs of Estonia<br>NCPHA, MoH Bulgaria<br>Permanent Representation of Italy to the International Organizations<br>The State Agency for Prevention of Alcohol Related Problems, Poland<br>United States of America<br>**UN system and other international organisations (IGOs)**<br>The Pacific Community (SPC) (on behalf of Pacific Island Countries and Territories)<br>UNDP<br>**Academic institutions**<br>Community Action on Youth and Drugs National Coordination Team, Massey University<br>MRC/CSO Social and Public Health Sciences Unit, University of Glasgow<br>School of Public Health, LKS Faculty of Medicine, The University of Hong Kong<br>SHORE Research Centre<br>SPECTRUM (Shaping Public hEalth poliCies To Reduce IneqUalities and harM)<br>TRAPS (Transformative Research on Alcohol Policy and Science programme at the University of York)<br>**Non-governmental organisations (NGOs)**<br>Abstinentenverband des Kantons Zürich<br>AESKAN<br>Alcohol & Drug Information Centre (ADIC), India

			Alcohol Action Ireland<br>Alcohol Action New Zealand<br>Alcohol and Drug Information Centre (ADIC)<br>Alcohol Focus Scotland<br>Alcohol Health Alliance<br>Amardeep India<br>APABurkina<br>Asia Pacific Alcohol Policy Alliance<br>Canadian Centre for Substance use and Addiction (CCSA)<br>Cancer Society<br>Centre for Alcohol Studies, Thai Health Promotion Foundation<br>Cruz Azul no Brasil<br>EHYT Finnish Association for Substance Abuse Prevention<br>European Alcohol Policy Alliance<br>FORUT<br>Foundation for Alcohol Research and Education<br>Global Alcohol Policy Alliance<br>Hāpai Te Hauora Tapui Limited<br>Healthy India Alliance<br>Hope and Beyond<br>HRIDAY<br>Humankind Charity<br>Institute for Research and Development "Utrip"<br>Institute of Alcohol Studies<br>International Federation of Medical Students' Association (IFMSA)<br>International Youth Health Organizations<br>IOGT Germany<br>IOGT Guinea-Bissau<br>IOGT Iceland<br>IOGT International<br>IOGT Norway<br>IOGT Switzerland<br>IOGT-NTO<br>Junis<br>Juvente<br>Juvente Switzerland<br>Kettil Bruun Society for Social and Epidemiological Research on Alcohol<br>Liberia Alcohol Policy Alliance<br>Lithuanian Tobacco and Alcohol Control Coalition<br>movendi slovakia<br>Nada India Foundation<br>NCD Alliance<br>Newcastle Coalition inner city resident groups, small businesses and concerned citizens<br>Nigeria Alcohol Prevention Youth Initiative<br>Nordic Alcohol and Drug Policy Network (NordAN)<br>Núll Prósent Hreyfingin

			Research and Training Center for Community Development (RTCCD)- The coordination organization of the Vienam Non-Communicable Diseases Control and Prevention Alliance (NCDs-VN)<br>Scottish Health Action on Alcohol Problems - SHAAP<br>Senegalese Alcohol Policy Alliance (SenAPA)<br>Sierra Leone Alcohol Policy Alliance (SLAPA)<br>Southern African Alcohol Policy Alliance<br>Students Campaign Against Drugs<br>The Wellbeing Initiative<br>United States Alcohol Policy Alliance (U.S.APA)<br>Vision for Alternative Development<br>WAAPA-BENIN/ Secrétariat (Initiative pour l'Education et le Contrôle du Tabagisme)<br>West African Alcohol Policy Alliance (WAAPA)<br>Youth against Alcoholism and Drug Dependency (YADD)<br>**Private sector entities**<br>Alcohol Awareness Foundation Ireland (trading as Drinkaware)<br>Alcohol Beverages Australia<br>AssoBirra<br>Australian Grape and Wine Inc. Australian Grape & Wine)<br>Beer Canada<br>Beer Institute<br>Brazilian Beer Trade Association (SINDICERV)<br>Bundesverband der Deutschen Spirituosen-Industrie und -Importeure e.V. (BSI)/Federal Association of the German Spirits Industry and Importers (BSI)<br>Caribbean Breweries Association (CBA)<br>CEEV, Comité européen des entreprises vins<br>Cerveceros de España<br>Cerveceros Latinoamericanos<br>CTA – Confederation of Business Associations of Mozambique<br>Distilled Spirits Council of the United Sates<br>Drinks Ireland<br>DrinkWise<br>Educ'alcool<br>FEDERACIÓN ESPAÑOLA DEL VINO (FEV)<br>Fédération des Exportateurs de Vins et Spiritueux de France (FEVS)<br>FIVS<br>Fundación de Investigaciones Sociales A.C. (Foundation of Social Research)<br>International Alliance for Responsible Drinking (IARD)<br>ISWAI International Spirits & Wine Association of India<br>Japan Spirits & Liqueurs Makers Association (JSLMA)<br>Mexican Chamber of the Tequila Industry<br>México's National Chamber of Beer and Malt<br>Regional Beverage Alcohol Alliance (RBAA)<br>Representantes-Importadores de Vinos y Licores Asociados (RIVLAS)<br>South African Liquor Brand owners Association

			Spirits New Zealand, New Zealand Winegrowers and the Brewers Association of New Zealand<br>spiritsEUROPE<br>STIVA (Foundation for responsible alcohol consumption)<br>The Brewers of Europe<br>The UK alcoholic drinks trade associations: British Beer & Pub Association, National Association of Cider Makers, Scotch Whisky Association and Wine and Spirit Trade Association<br>Trinidad & Tobago Beverage Alcohol Alliance (TTBAA)<br>Vinos de Chile<br>West Indies Rum & Spirits Producers Association (WIRSPA)<br>World Spirits Alliance<br>Worldwide Brewing Alliance
	To oppose others' problem definitions, causes and solutions	"The relation between Per Capita consumption or Prevalence and Harmful Consumption cannot be directly linked, more studies are required. In general, there is just no single answer" (Cerveceros Latinoamericanos, trade association)<br><br>"According to a comprehensive review of advertising studies, longitudinal studies claiming to show a causal link between alcohol ads and youth drinking are scientifically flawed.28 This review found "significant econometric and statistical problems, which preclude a causal interpretation."29 Among the studies' flaws, the author reported problems with how researchers selected people to participate in their studies and how they drew conclusions from the data they collected.30" (Distilled Spirits Council of the United Sates, trade association)<br>**[Studies referenced have received funding from ICAP, an industry-funded social aspect organisation]**	**Member States and governmental institutions**<br>Permanent Representation of Italy to the International Organizations<br>United States of America<br>**UN system and other IGOs**<br>**Academic institutions**<br>SHORE Research Centre<br>SPECTRUM (Shaping Public hEalth poliCies To Reduce IneqUalities and harM)<br>TRAPS (Transformative Research on Alcohol Policy and Science programme at the University of York)<br>**NGOs**<br>Alcohol Health Alliance<br>APABurkina<br>Asia Pacific Alcohol Policy Alliance<br>Centre for Alcohol Studies, Thai Health Promotion Foundation<br>FORUT<br>Foundation for Alcohol Research and Education<br>Global Alcohol Policy Alliance<br>Institute of Alcohol Studies<br>Liberia Alcohol Policy Alliance<br>Nordic Alcohol and Drug Policy Network (NordAN)<br>Research and Training Center for Community Development (RTCCD)- The coordination organization of the Vienam Non-Communicable Diseases Control and Prevention Alliance (NCDs-VN)<br>Senegalese Alcohol Policy Alliance (SenAPA)<br>Sierra Leone Alcohol Policy Alliance (SLAPA)<br>Southern African Alcohol Policy Alliance<br>Students Campaign Against Drugs<br>WAAPA-BENIN/ Secrétariat (Initiative pour l'Education et le Contrôle du Tabagisme)<br>West African Alcohol Policy Alliance (WAAPA)<br>**Private sector entities**<br>Alcohol Beverages Australia<br>Beer Canada<br>Brazilian Beer Trade Association (SINDICERV)

			Caribbean Breweries Association (CBA)<br>Cerveceros Latinoamericanos<br>CTA – Confederation of Business Associations of Mozambique<br>Distilled Spirits Council of the United Sates<br>Educ'alcool<br>Fédération des Exportateurs de Vins et Spiritueux de France (FEVS)<br>FIVS<br>Fundación de Investigaciones Sociales A.C. (Foundation of Social Research)<br>International Alliance for Responsible Drinking (IARD)<br>Mexican Chamber of the Tequila Industry<br>Regional Beverage Alcohol Alliance (RBAA)<br>South African Liquor Brand owners Association<br>Spirits New Zealand, New Zealand Winegrowers and the Brewers Association of New Zealand<br>spiritsEUROPE<br>The UK alcoholic drinks trade associations: British Beer & Pub Association, National Association of Cider Makers, Scotch Whisky Association and Wine and Spirit Trade Association<br>Trinidad & Tobago Beverage Alcohol Alliance (TTBAA)<br>Vinos de Chile<br>Worldwide Brewing Alliance
**Tobacco analogies**	Alcohol control requires a similar approach to tobacco control	"Since alcohol's adverse effects on the individual and society are bigger than those of tobacco, the frame of NCDs in which alcohol is currently located, contributes to the distortion of the understanding of alcohol harm in its full extent." (IOGT, NGO)<br><br>"Alcohol, food and drink industries are known to use similar tactics to the tobacco industry to undermine effective public health policies and interventions." (Foundation for Alcohol Research and Education, NGO)<br><br>"In line with what happens with tobacco, in article 5.3 of the FCTC, and in recognition of the inherent conflicts of interest that exist, there need to be strict rules prescribing the types of engagement permitted between states and international agencies on the one hand and economic operators/the liquor industry on the other."	**Member States and governmental institutions**<br>Cook Islands Ministry of Health<br>Directorate of Health, Iceland<br>Ministry of Health of the Republic of Latvia<br>Ministry of Health, National Commission Against Addictions, Mexico<br>Ministry of Public Health, Thailand<br>Ministry of Social Affairs of Estonia<br>South African Medical Research Council<br>**UN system and other IGOs**<br>The Pacific Community (SPC) (on behalf of Pacific Island Countries and Territories)<br>UNDP<br>**Academic institutions**<br>Community Action on Youth and Drugs National Coordination Team, Massey University<br>MRC/CSO Social and Public Health Sciences Unit, University of Glasgow<br>School of Public Health, LKS Faculty of Medicine, The University of Hong Kong<br>SHORE Research Centre<br>SPECTRUM (Shaping Public hEalth poliCies To Reduce IneqUalities and harM)<br>TRAPS (Transformative Research on Alcohol Policy and Science programme at the University of York)<br>**NGOs**<br>Abstinentenverband des Kantons Zürich<br>AESKAN<br>Alcohol & Drug Information Centre (ADIC)-India<br>Alcohol and Drug Information Centre (ADIC)<br>Alcohol Focus Scotland

		(South African Medical Research Council, Member State)	Alcohol Health Alliance<br>Amardeep India<br>Australasian Professional Society on Alcohol and other Drugs (APSAD)<br>Balance, the North East Alcohol Office<br>Canadian Centre for Substance use and Addiction (CCSA)<br>Cancer Society<br>Centre for Alcohol Studies, Thai Health Promotion Foundation<br>Fondacioni YESILAY<br>FORUT<br>Foundation for Alcohol Research and Education<br>Foundation for Innovative Social Development (FISD)<br>Global Alcohol Policy Alliance<br>HealthBridge Foundation of Canada, Vietnam Office<br>Healthy India Alliance<br>Hong Kong Alliance for Advocacy Against Alcohol<br>Hope and Beyond<br>HRIDAY<br>Institute for Research and Development "Utrip"<br>Institute of Alcohol Studies<br>International Blue Cross<br>International Federation of Medical Students' Association (IFMSA)<br>International Youth Health Organizations<br>IOGT Guinea-Bissau<br>IOGT International<br>IOGT Norway<br>IOGT Switzerland<br>IOGT-NTO<br>Junis<br>Juvente<br>Juvente Switzerland<br>Kettil Bruun Society for Social and Epidemiological Research on Alcohol<br>Lithuanian Tobacco and Alcohol Control Coalition<br>movendi slovakia<br>Nada India Foundation<br>NCD Alliance<br>Nigeria Alcohol Prevention Youth Initiative<br>Núll Prósent Hreyfingin<br>Public Union against Bad Habits<br>Research and Training Center for Community Development (RTCCD)- The coordination organization of the Vienam Non-Communicable Diseases Control and Prevention Alliance (NCDs-VN)<br>Southern African Policy Alliance<br>Sri Lanka Medical Association<br>The Wellbeing Initiative<br>Trimbos Institute<br>Vision for Alternative Development

			Youth against Alcoholism and Drug Dependency (YADD)
**Value systems**	Neoliberal values: Individuals have the right to consume alcohol, and alcohol harms should be addressed by the private sector	"We need to recognise that encouraging responsible consumption will reduce alcohol related harm and the associated health and externality costs. History shows that prohibition drives illicit consumption, health related risk and increased abuse. We require a proportionate response." (Australian Grape and Wine Inc., trade association)<br><br>"In the United States, commercial speech and the right to advertise are constitutionally protected under the First Amendment. Such First Amendment protection afforded to beverage alcohol advertising is equal in scope to the First Amendment protection afforded to the advertising of other legal products and services." (Distilled Spirits Council of the United States, trade association)<br><br>"The United States disagrees with implied blanket criticisms of international, regional and bilateral trade agreements in the Discussion Paper. Countries carefully consider agreements into which they enter; it is not the role of international organizations to cast aspersions on the sovereign decisions of Member States in this regard." (United States of America, Member State)	**Member States and governmental institutions**<br>Guyana Mission<br>Ministry of agriculture, Latvia<br>Permanent Representation of Italy to the International Organizations<br>United States of America<br>**UN system and other IGOs**<br>United Nations Conference on Trade and Development<br>**Private sector entities**<br>Alcohol Awareness Foundation Ireland (trading as Drinkaware)<br>Alcohol Beverages Australia<br>Asociación Dominicana de Productores de Ron (ADOPRON)<br>AssoBirra<br>Association for Alcohol Responsibility and Education (aware.org)<br>Association of Alcohol Manufacturers and Importers<br>Australian Grape and Wine Inc. Australian Grape & Wine)<br>Beer Canada<br>Beer Institute<br>Belgian Brewers<br>Brazilian Beer Trade Association (SINDICERV)<br>Bundesverband der Deutschen Spirituosen-Industrie und -Importeure e.V. (BSI)/Federal Association of the German Spirits Industry and Importers (BSI)<br>Caribbean Breweries Association (CBA)<br>CEEV, Comité européen des entreprises vins<br>Cerveceros de España<br>Cerveceros Latinoamericanos<br>CTA – Confederation of Business Associations of Mozambique<br>Distilled Spirits Council of the United Sates<br>Drinks Ireland<br>DrinkWise<br>Educ'alcool<br>FEDERACIÓN ESPAÑOLA DEL VINO (FEV)<br>Fédération des Exportateurs de Vins et Spiritueux de France (FEVS)<br>FIVS<br>Fundación de Investigaciones Sociales A.C. (Foundation of Social Research)<br>International Alliance for Responsible Drinking (IARD)<br>ISWAI International Spirits & Wine Association of India<br>Japan Spirits & Liqueurs Makers Association (JSLMA)<br>Mexican Chamber of the Tequila Industry<br>México's National Chamber of Beer and Malt<br>Regional Beverage Alcohol Alliance (RBAA)<br>Representantes-Importadores de Vinos y Licores Asociados (RIVLAS)<br>South African Liquor Brand owners Association<br>Spirits New Zealand, New Zealand Winegrowers and the Brewers Association of New Zealand<br>spiritsEUROPE

			STIVA (Foundation for responsible alcohol consumption)<br>The Brewers of Europe<br>The UK alcoholic drinks trade associations: British Beer & Pub Association, National Association of Cider Makers, Scotch Whisky Association and Wine and Spirit Trade Association<br>Trinidad & Tobago Beverage Alcohol Alliance (TTBAA)<br>Vinos de Chile<br>West Indies Rum & Spirits Producers Association (WIRSPA)<br>World Spirits Alliance<br>Worldwide Brewing Alliance
	Human rights values: Alcohol and its harms violate basic human right and should be regulated against	"The Global Alcohol Strategy does not contain a reference to the link between alcohol and human rights or relevant human rights instruments. We support the inclusion of reference to relevant human rights and human rights instruments such as for example Article 12 of the International Covenant on Economic, Social and Cultural Rights, adopted by the United Nations General Assembly on 16 December 1966, which states that it is the right of everyone to the enjoyment of the highest attainable standard of physical and mental health (…)." (McCabe Centre for Law & Cancer, NGO)<br><br>"The implementation of Decree 9.761 / 2019 should consider that Brazil is a signatory to the International Convention on the Rights of the Child, which has the power of the Federal Constitution. Effective actions will be necessary to ensure the rights of children and adolescents, aiming at their integral health and to protect them from products harmful to their health, such as alcohol." (Cruz Azul no Brasil, NGO)<br><br>"The acceleration of trade and investment agreements around the world require urgent responses to protect governments' rights to regulate the alcohol industry and	**Member States and governmental institutions**<br>Centre for Diseases Prevengtion and Control, Latvia<br>Cook Islands Ministry of Health<br>Department of Health, Ireland<br>Directorate of Health, Iceland<br>Federal Office for Public Health, Switzerland<br>FPS Public health, Food chain safety and Environment, Belgium<br>Instituto sobre Alcoholismo y Fármacodependencia (IAFA), Costa Rica<br>Ministerio de Salud Pública de la República de Cuba<br>Ministry of Health Mozambique<br>Ministry of Health of the Czech Republic<br>Ministry of Health of the Republic of Latvia<br>Ministry of Health, National Commission Against Addictions, Mexico<br>Ministry of Health, New Zealand<br>Ministry of Health, Republic of Slovenia<br>Ministry of Health, Welfare and Sport, The Netherlands<br>Ministry of Public Health, Thailand<br>Ministry of Social Affairs of Estonia<br>NCPHA, MoH Bulgaria<br>Permanent Mission of Georgia to the United Nations Office in Geneva and other international organizations<br>South African Medical Research Council<br>Spanish Ministry of Health, Consumer Affairs and Welfare<br>The National Institute of Public Health, Czech Republic<br>The State Agency for Prevention of Alcohol Related Problems, Poland<br>**UN system and other IGOs**<br>European Centre Social Welfare Policy and Research<br>The Pacific Community (SPC) (on behalf of Pacific Island Countries and Territories)<br>UNDP<br>**Academic institutions**<br>Community Action on Youth and Drugs National Coordination Team, Massey University<br>MRC/CSO Social and Public Health Sciences Unit, University of Glasgow<br>School of Public Health, LKS Faculty of Medicine, The University of Hong Kong<br>SHORE Research Centre<br>SPECTRUM (Shaping Public hEalth poliCies To Reduce IneqUalities and harM)

		to legislate in the area of alcohol policy." (IOGT, NGO)	TRAPS (Transformative Research on Alcohol Policy and Science programme at the University of York)<br>**NGOs**<br>Abstinentenverband des Kantons Zürich<br>AESKAN<br>AFGHANISTAN GREEN CRESCENT ORGANIZAION (AGCO)<br>Alcohol & Drug Information Centre (ADIC), India<br>Alcohol Action Ireland<br>Alcohol Action New Zealand<br>Alcohol and Drug Information Centre (ADIC)<br>Alcohol Focus Scotland<br>Alcohol Health Alliance<br>Alcohol Policy Alliance Gambia<br>Amardeep India<br>APABurkina<br>Asia Pacific Alcohol Policy Alliance<br>Association for Promoting Social Action (APSA)<br>Association of Advocates against Alcohol Harm in Nigeria<br>Australasian Professional Society on Alcohol and other Drugs (APSAD)<br>Balance, the North East Alcohol Office<br>Canadian Centre for Substance use and Addiction (CCSA)<br>Cancer Society<br>Center for youth education<br>Centre for Alcohol Studies, Thai Health Promotion Foundation<br>CROISSANT VERT NIGERIEN(CVN)<br>Cruz Azul no Brasil<br>EHYT Finnish Association for Substance Abuse Prevention<br>European Alcohol Policy Alliance<br>European Mutual help Network for Alcohol related problems (EMNA)<br>Fondacioni YESILAY<br>FORUT<br>Foundation for Alcohol Research and Education<br>Foundation for Innovative Social Development (FISD)<br>Global Alcohol Policy Alliance<br>Green crescent of Congo est<br>Green Crescent Society, Turkey<br>Green Crescent South Africa<br>Green Crescent Zimbabwe<br>Hāpai Te Hauora Tapui Limited<br>HealthBridge Foundation of Canada, Vietnam Office<br>Healthy India Alliance<br>Hong Kong Alliance for Advocacy Against Alcohol<br>Hope and Beyond<br>HRIDAY<br>Humankind Charity<br>Institute for Research and Development "Utrip"

			Institute of Alcohol Studies<br>International Blue Cross<br>International Federation of Medical Students' Association (IFMSA)<br>International Youth Health Organizations<br>IOGT Gambia<br>IOGT Germany<br>IOGT Guinea-Bissau<br>IOGT Iceland<br>IOGT International<br>IOGT Norway<br>IOGT Switzerland<br>IOGT-NTO<br>Italian Society on Alcohol (SIA)<br>Junis<br>Juvente<br>Juvente Switzerland<br>Kettil Bruun Society for Social and Epidemiological Research on Alcohol<br>Liberia Alcohol Policy Alliance<br>Lithuanian Tobacco and Alcohol Control Coalition<br>McCabe Centre for Law & Cancer<br>Moroccan Green Crescent<br>movendi slovakia<br>Nada India Foundation<br>National Alliance for Action on Alcohol<br>NCD Alliance<br>Newcastle Coalition inner city resident groups, small businesses and concerned citizens<br>NGO “ Zeleni krst - Zeleni polumjesec” - Serbia<br>Nigeria Alcohol Prevention Youth Initiative<br>Nordic Alcohol and Drug Policy Network (NordAN)<br>Núll Prósent Hreyfingin<br>People Center for Development and Peace<br>Pioneer Total Abstinence Association<br>Recovery And Humanitarian Action Management Agency (RAHAMA)<br>RECOVERY, z.s.<br>Research and Training Center for Community Development (RTCCD)- The coordination organization of the Vienam Non-Communicable Diseases Control and Prevention Alliance (NCDs-VN)<br>Scottish Health Action on Alcohol Problems - SHAAP<br>Senegalese Alcohol Policy Alliance (SenAPA)<br>Serenity Harm Reduction Programme Zambia (SHARPZ)<br>Sierra Leone Alcohol Policy Alliance (SLAPA)<br>Southern African Alcohol Policy Alliance<br>Sri Lanka Medical Association<br>Stopdrink Network<br>Students Campaign Against Drugs<br>Swedish cancer society

			Tanzania Media Women's Association (TAMWA)<br>Tanzania Network Against Alcohol Abuse – TAAnet<br>The Wellbeing Initiative<br>Trimbos Institute<br>UDRUZENJE GRADANA ZELENI POLUMJESEC U BIH<br>Uganda Youth Development Link<br>UNF<br>United States Alcohol Policy Alliance (U.S.APA)<br>value health africa<br>Vision for Alternative Development<br>WAAPA-BENIN/ Secrétariat (Initiative pour l'Education et le Contrôle du Tabagisme)<br>West African Alcohol Policy Alliance (WAAPA)<br>World Federation Against Drugs<br>Youth against Alcoholism and Drug Dependency (YADD)
